# Imputation of Unordered Markers and the Impact on Genomic Selection Accuracy

**DOI:** 10.1534/g3.112.005363

**Published:** 2013-03-01

**Authors:** Jessica E. Rutkoski, Jesse Poland, Jean-Luc Jannink, Mark E. Sorrells

**Affiliations:** * Department of Plant Breeding and Genetics, Cornell University, Ithaca New York, 14853; † Department of Agronomy, Kansas State University, Manhattan, Kansas 66506; ‡ United States Department of Agriculture-Agricultural Research Service (USDA-ARS), Manhattan, Kansas 66502; § USDA-ARS, Ithaca, New York 14853

**Keywords:** genomic selection, imputation algorithms, genotyping-by-sequencing, GenPred, Shared data resources

## Abstract

Genomic selection, a breeding method that promises to accelerate rates of genetic gain, requires dense, genome-wide marker data. Genotyping-by-sequencing can generate a large number of *de novo* markers. However, without a reference genome, these markers are unordered and typically have a large proportion of missing data. Because marker imputation algorithms were developed for species with a reference genome, algorithms suited for unordered markers have not been rigorously evaluated. Using four empirical datasets, we evaluate and characterize four such imputation methods, referred to as k-nearest neighbors, singular value decomposition, random forest regression, and expectation maximization imputation, in terms of their imputation accuracies and the factors affecting accuracy. The effect of imputation method on the genomic selection accuracy is assessed in comparison with mean imputation. The effect of excluding markers with a large proportion of missing data on the genomic selection accuracy is also examined. Our results show that imputation of unordered markers can be accurate, especially when linkage disequilibrium between markers is high and genotyped individuals are related. Of the methods evaluated, random forest regression imputation produced superior accuracy. In comparison with mean imputation, all four imputation methods we evaluated led to greater genomic selection accuracies when the level of missing data was high. Including rather than excluding markers with a large proportion of missing data nearly always led to greater GS accuracies. We conclude that high levels of missing data in dense marker sets is not a major obstacle for genomic selection, even when marker order is not known.

Genomic selection (GS) (Meuwissen *et al.* 2001) is a relatively new breeding methodology reviewed by Hayes *et al.* (2009), Heffner *et al.* (2009), and Lorenz *et al.* (2011) that is increasingly attractive for the genetic improvement of various species because of its potential to increase the rate of genetic gain (Wong and Bernardo 2008; Lorenzana and Bernardo 2009; Heffner *et al.* 2010). With GS, a training population having both phenotypic data and genome-wide marker data is used to develop a prediction model for the trait of interest. Before phenotyping, this prediction model is then applied to selection candidates that have been genotyped. Genomic-estimated breeding values are calculated for the selection candidates and selections are made using these values. These breeding values are estimated using genotypes instead of phenotypes; therefore, selection can occur in early stages on a single plant basis or in situations in which phenotyping is either not possible, unreliable, or too expensive, thus leading to shorter selection cycles.

One of the requirements for GS is genome-wide marker coverage. In general, one marker should be in linkage disequilibrium (LD) with each segregating segment of the genome. The choice of marker platform is driven by the available genotyping technology and the cost per data-point. Genotyping-by-sequencing (GBS) is gaining popularity because it can be less expensive than other platforms and can provide genome-wide marker coverage for species that lack genotyping resources such as pre-designed single-nucleotide polymorphism platforms (Poland and Rife 2012). Polymorphic loci scored by GBS can contain a large proportion of missing data across samples because random fragments of the genome are sequenced at low depth, leading some loci to have zero coverage in some individuals (Elshire *et al.* 2011). The proportion of missing data depends on the sequencing depth and library complexity. Greater sequencing depth leads to a smaller proportion of missing data but increases genotyping cost. Less-complex libraries, on the other hand, will have less missing data but a fewer markers. To generate a large number of markers at low cost, low sequencing depth is commonly used, leading to a large proportion of missing data points. Most analyses require a complete dataset; therefore, marker imputation is a necessary step before GBS data can be used for most purposes.

Imputation has been shown to increase power in association mapping studies (Marchini *et al.* 2007; Marchini and Howie 2010) and, for GS, imputation can enable the use of low-density genotyping without a major loss in accuracy because a closely related reference panel genotyped at high density can be used to impute markers not present in the low-density marker panel. (Habier *et al.* 2009; Weigel *et al.* 2010; Dassonneville *et al.* 2011; Mulder *et al.* 2012). Although several highly accurate and widely used imputation algorithms have been developed to assign allelic states of missing values in genotype data (reviewed by Pei *et al.* 2008 and Marchini *et al.* 2010), these algorithms were designed for human genetic data, and they require that the order of the markers be known because they are based on constructing haplotypes. For species lacking a reference genome and complete reference linkage map such as wheat, *Triticum aestivum* L., the majority of markers typed on a given population are unordered, and current genotype imputation methods cannot be used. Although for biparental populations linkage maps can be constructed, breeding populations for GS are derived from multiple parents and not well structured for developing genetic maps. Thus, alternative imputation strategies that are map-independent are necessary when GBS is used for species lacking a reference genome sequence and for populations unsuitable for linkage map construction. There are many general imputation methods that do not require any prior information about the variables to be imputed. Although these methods are used across many disciplines, they have not been tested for imputation accuracy of genome-wide marker data. It is also not known how imputation with a general and potentially less accurate method before GS model training will affect the GS model accuracy. However, we expect these imputation methods to improve the GS accuracy because during the imputation step, genotypic information from both the training and selection sets is used to estimate missing values. Thus, the validation set helps improve imputation of the training set and vice versa.

The objective of this study was to evaluate imputation strategies that do not require previous information about the order of the markers. The imputation methods compared were: mean imputation (MNI), k-nearest neighbors imputation (kNNI) (Troyanskaya *et al.* 2001), singular value decomposition imputation (SVDI) (Troyanskaya *et al.* 2001), expectation maximization imputation (EMI) (Dempster *et al.* 1977), and random forest regression imputation (RFI) (Stekhoven and Bühlmann 2011). By using array-based genotypic datasets with varying levels of simulated missing data, we compared these methods in terms of their imputation accuracy, computation time, and impact on GS prediction accuracy. The factors affecting imputation accuracy for each method at the marker genotype and individual genotype level were also examined. Finally, we determined whether excluding rather than including markers with high levels of missing data could lead to higher accuracy.

## Materials and Methods

### Original datasets

We used five different datasets consisting of genome-wide markers and breeding value estimates. These datasets are referred to as winter wheat (WW), spring wheat (SW), drought tolerant maize (DTM), North American barley (NAB), and stem rust resistant wheat (SRRW). The WW data (Supporting Information, File S2) consists of 374 elite inbred individuals originating from the Cornell winter wheat breeding program. The markers consisted of 1158 polymorphic diversity array technology (DArT) (Akbari *et al.* 2006) markers coded as “−1” and “1.” For a more detailed description of this dataset, refer to Heffner *et al.* (2011). The traits used for the evaluation of cross-validated GS accuracies for WW were grain yield, height (HT), protein, and days to heading. The SW data are a historical dataset consisting of 599 elite inbred spring wheat lines originating from the International Maize and Wheat Improvement Center (CIMMYT) wheat breeding program. The markers consist of 1279 polymorphic DArT markers coded as “0” and “1” and the trait used for the evaluation of cross-validated GS accuracies was grain yield in CIMMYT mega-environment 1. The DTM data consists of 264 tropical CIMMYT maize lines. The trait used to calculate cross-validated GS model accuracies for DTM was grain yield. The marker data consists of 1135 single-nucleotide polymorphisms coded as “−1”, “0”, and “1.” For more details about the SW and DTM datasets, or to access these datasets, refer to Crossa *et al.* (2010). The NAB dataset consists of a North American spring barley association mapping panel evaluated from 2006 to 2008 as part of the Barley Coordinated Agricultural Project (2011). The panel consists of 911 individuals with 2146 polymorphic single-nucleotide polymorphisms. The trait used to calculate GS model accuracies was beta-glucan content (B-glucan). The data can be accessed at http://triticeaetoolbox.org/barley.

The SRRW dataset consists of 360 recent, elite CIMMYT spring wheat lines that have been selected for quantitative resistance to stem rust caused by *Puccinia graminis* f.sp. *tritici*. The markers consist of more than 130,000 GBS polymorphisms. Three different versions of the SRRW GBS data, described in Table 1, were created based on different per-marker percent missing data thresholds. For the first version referred to as SRRW version NA20 (File S3), markers were excluded if they had more than 20% missing values, which resulted in 2014 total markers. For the second set and third sets, referred to as SRRW versions NA50 and NA70 (File S4 and File S5), markers were excluded if they had more than 50% and 70% missing data, respectively, and then 2014 markers were randomly selected. The percent of the data points that were missing in the original WW, SW, DTM, and NAB datasets was between 0.2 and 3%. This low level of pre-existing missing data was assumed to have a negligible effect on the imputation and GS accuracies and for these datasets the original marker data are referred to as version NA0.

**Table 1:** Description of datasets used for imputation and genomic selection

Dataset	Version*^a^*	Mean Percent Missing Data Points*^b^*	Number of Markers	Number of Individuals
	NA20	12.13	1158	374
WW	NA50	34.08	1158	374
	NA70	58.84	1158	374
	NA20	12.1	1279	599
SW	NA50	34.98	1279	599
	NA70	60.54	1279	599
	NA20	11.99	1135	264
DTM	NA50	34.9	1135	264
	NA70	60.53	1135	264
	NA20	12.1	2146	911
NAB	NA50	35.03	2146	911
	NA70	60.49	2146	911
	NA20	12.16	2014	360
SRRW	NA50	35.13	2014	360
	NA70	60.72	2014	360

WW, Cornell winter wheat; SW, CIMMYT elite spring wheat; DTM, CIMMYT drought-tolerant maize; NAB, North American barley; SRRW, CIMMYT stem rust-resistant wheat.

a NA20: up to 20% missing data per marker, NA50: up to 50% missing data per marker, NA70: up to 70% missing data per marker.

b The percent of total data points that are missing.

### Calculation of LD between marker pairs

For the original WW, SW, DTM, and NAB datasets, LD between all marker pairs was measured using the r^2^ statistic, where r^2^ between two markers was calculated using the formula:

$${\text{r}}^{2}=\frac{D^{2}}{p_{1}q_{1}p_{2}q_{2}}$$

where *D* = *x*_11_ -*p*_1_*p*_2_; *x*_11_is the probability of observing the combination of allele 1 at marker *j* and allele 1 at marker *l*, *p*_1_ is the probability of allele 1 at marker *j*, *q*_1_ is the probability of allele 2 at marker *j*, *p*_2_ is the probability of allele 1 at marker *l*, and *q*_2_ is the probability of allele 2 at marker *l*. A maximum likelihood estimate of *x*_11_ was obtained using an expectation maximum approach reviewed by Foulkes (2009). All calculations of the r^2^ statistic were implemented in the R package “genetics” (Warnes *et al.* 2011).

### Missing data simulation

For each of the WW, SW, DTM, and NAB datasets three versions of the genotypic data, summarized in Table 1, were created with different levels of simulated missing data. In each of the versions: NA20, NA50, and NA70, missing values were introduced at random but the maximum percent missing data at a given marker was set to 20%, 50%, and 70% respectively. Examples of the simulated markers sets are illustrated in the Figure S1. A total of 10 replicates of each simulated dataset were created, and the mean percent of total data points that are missing across the 10 replicates is shown in Table 1. The distribution of per-marker percent missing values from the SRRW data versions NA20, NA50, and NA70 were used to assign the percent missing at each marker for each of the WW, SW, DTM, and NAB datasets to produce versions NA20, NA50, and NA70, respectively. Across all the missing data versions of all the datasets, the percent missing per marker distribution had a long left tail and a large concentration of values near the threshold level.

### Imputation methods

In all cases, the genotypic data were considered continuous variables. The methods MNI, kNNI, SVDI, EMI, and RFI were used to impute the simulated missing values. For all methods the input was an *m* x *n* genotype matrix M with *m* individuals and *n* markers. For MNI, each missing data-point *x_ij_* at a given marker *j* was replaced with the mean of the non-missing values at that marker.

For kNNI (Troyanskaya *et al.* 2001), the data points were imputed by replacing them with the weighted average of the data points at the k closest markers. Euclidean distance was used as the measure of marker distance. Euclidean distance between marker genotype vectors $\vec{q}$ and $\vec{v}$ of length *m* was defined as*:*

$$d\left(\vec{q},\vec{v}\right)=\sqrt{{\left(q_{1}-v_{1}\right)}^{2}+{\left(q_{2}-v_{2}\right)}^{2}+\cdot \cdot \cdot +{\left(q_{m}-v_{m}\right)}^{2}}$$

In detail, (1) missing values were first replaced using MNI and the Euclidean distance between all of possible pairs of marker vectors was computed. Each marker was included in the marker matrix twice, both in its original and flipped state, to ensure that markers in negative LD would not be considered distant to the marker of interest. (2) For each marker *j*, markers were sorted based on Euclidean distance to marker *j*. (3) For each row *i* of marker *j* the weighted average of the k closest markers with nonmissing values at row *i* were used as an estimate of marker data point *x_ij_*. The weight of each marker was 1/*d^2^* were *d* is the Euclidean distance between marker *j* and the marker to be weighted. kNNI makes no assumptions about the distribution of the data.

For SVDI (Troyanskaya *et al.* 2001), a singular value decomposition of genotype matrix M was used to obtain a set of the k most significant Eigen-vectors of the markers. These k Eigen-vectors were then used as the predictors for linear regression estimation of the missing data points. SVDI was implemented in R (R Development Core Team 2011) using the package “bcv” (Perry 2009). The genotype matrix M can be described as:

$$\text{M}=\text{U}\sum V^{T}$$

Where U has dimensions *m* × k, V has dimensions *n* × k, and Σ is a k × k diagonal matrix. U contains the left singular vectors with are equivalent to the Eigen-vectors of the markers. The corresponding singular values are in the diagonal elements of Σ. The singular values are equivalent to the square root of the Eigen-values. The k most significant Eigen-vectors of the markers were those with the k largest Eigen-values. The imputation procedure is described as follows: (1) Missing values were originally imputed using MNI. (2) Singular value decomposition was used to estimate the k most significant Eigen-vectors of the markers: $\hat{\text{U}}$. (3) For each marker *j*, linear regression coefficients of each column of $\hat{\text{U}}$ were estimated by the multiple linear regression equation:

$$\text{Y}=\hat{U}\beta +\varepsilon$$

Where Y is a column vector for marker *j*, $\hat{\text{U}}$ is an *m* × *k* matrix of k Eigen-vectors, β is a vector of regression coefficients and ε is a random error term. Only individuals with nonmissing values in Y were used to estimate β. (4) $\hat{\text{U}}$ and the estimates of the regression coefficients, $\hat{\beta }$, were used to estimate the missing values at marker *j*. (5) Using the current version of the genotype matrix, we repeated steps two through four for a total of 10 iterations, sufficient to meet the convergence criteria, which was:

$$\frac{\left|RSS_{0}-RSS_{1}\right|}{RSS_{1}}<0.02$$

*RSS* is the residual sum of squares between the nonmissing values and their SVDI model approximation. *RSS*_0_ and *RSS*_1_ are the *RSS* values of successive iterations. SVDI assumes that the genotype matrix is multivariate normal distributed. For the optimal k value calculation methods and results for both kNNI and SVDI, see File S1. Optimal k values are listed in Table S1.

For EMI, the nonmissing marker data were used to obtain maximum likelihood estimates of the vector of means, $\hat{u}$, and covariance matrix $\hat{X}$ of the individuals based on the markers. These estimates were then used to obtain multiple linear regression estimates of the missing marker values. $\hat{u}$, and $\hat{X}$ were then re-estimated and were used to re-estimate the missing marker values. This process was repeated until the difference between the new estimate and the previous estimate of $\hat{u}+\hat{X}*{\hat{X}}^{T}$ was 0.02 or less. EMI was implemented using the R package rrBLUP (Endelman 2011). For a more detailed description of this EMI algorithm, refer to Poland *et al.* (2012). For a more through description of the EM imputation algorithm in general, refer to Dempster *et al.* (1977).

For RFI, missing marker values were estimated using random forest regression (Breiman 2001) using all available data to predict the missing values for every marker. RFI was implemented in R using the package “MissForest” (Stekhoven and Bühlmann 2011). The imputation procedure was: (1) for marker matrix M, markers were sorted from lowest to highest percent missing and missing values were imputed using MNI. (2) At each marker *j* containing missing values, the nonmissing values, Y, were used to grow 100 random forest regression trees Θ1… Θ100. Each tree was grown using a bootstrapped sample of individuals Y and a random sample of $\sqrt{n-1\;}$marker predictors were used where *n*-1 is the number of markers excluding marker *j*. Each tree Θ contains the terminal node values and a set of instructions for recursively partitioning the observations into the terminal nodes: these instructions include the split variables at each node, and the value of the split variable used for partitioning. (3) Missing values at marker *j* were imputed as:

$$\hat{Y}=\frac{1}{100}\sum_{1}^{100}h\left(x,\Theta \right)$$

where *x* is an input vector.

(4) Marker *j* was then updated in marker matrix M by using the $\hat{Y}$ values as the estimate of the missing values. (5) Steps two through four were repeated for each subsequent marker until all markers were imputed. (6) Then, using this imputed matrix, we repeated steps two through five until convergence or for a maximum of 10 iterations. Convergence was declared as soon as the Δ*N* increased for the first time where:

$$\Delta N=\frac{\sum_{j\varepsilon n}{\left({\text{M}}_{1}-{\text{M}}_{0}\right)}^{2}}{\sum_{j\varepsilon n}{\left({\text{M}}_{1}\right)}^{2}}$$

M_1_ and M_0_ are the newly imputed and previously imputed marker matrices respectively. If the convergence criterion was met, M_0_ was used as the final estimate of M. RFI makes no assumptions about the distribution of the data. The implementation of all imputation methods is demonstrated in File S6.

### Imputation accuracy calculations

The per-marker imputation accuracy, $R_{m}^{2}$, was described using the *R*^2^ value between predicted data points and the original data points for a given marker vector or individual vector *x* of length *j*. The *R*^2^ was defined as

$$R^{2}=1-\frac{\sum_{j}{\left(xj\;\text{true}-xj\;\text{imputed}\right)}^{2}}{\sum_{j}{\left(xj\;\text{true}-\text{mean}(x)\right)}^{2}}$$

The $R_{m}^{2}$, as well as the imputation *R*^2^ of the individual genotypes, referred to as $R_{i}^{2}$, were calculated. For each dataset and missing data level, average $R_{i}^{2}$ and $R_{m}^{2}$ across the 10 missing data simulations were also calculated and referred to as $\overset{–}{R_{i}^{2}}$ and $\overset{–}{R_{m}^{2}}$.

To compare with imputation accuracies reported in other publications, for each $\overset{–}{R_{m}^{2}}$ value, the equivalent percent correct was also calculated. Because imputed values were continuous, the percent correct for each marker could not be directly calculated. Instead, for each marker, equivalent percent correct values were determined by simulation using each marker’s MAF and $\overset{–}{R_{m}^{2}}$ (see File S1).

### Computational time

For the first replicate of simulated missing datasets, whenever a dataset was imputed, the number of seconds required for imputation to be completed using one central processing unit was recorded. All jobs were submitted to the Computational Biology Service Unit at Cornell University, which uses (1) a 240 core Windows cluster consisting of 60 Dell PowerEdge 1855 nodes with two x64 Pentium 4 Xeon 3.4 GHz, 4 GB RAM, and 144 GB HD each and (2) a 400 core Windows cluster consisting of 200 Sun V20Z nodes with two AMD Opteron 248 2.2GHz, 2 GB RAM, and 300 GB HD each.

### Assessment of factors affecting imputation accuracy

For each imputation method factors affecting the imputation accuracy were assessed. A marker’s minor allele frequency (MAF), number of nonmissing data points, and level of LD with other markers were considered as factors that could impact its imputation accuracy. The distance between an individual and its closest relative and the expected prediction error variance (PEV) were considered as factors affecting the imputation accuracy on an individual genotype basis. The impact of each of these factors was assessed for each imputation method using the WW, SW, DTM, and NAB datasets post imputation.

First, the impact of MAF on the imputation accuracy was assessed. For each dataset-imputation method combination, $\overset{–}{R_{m}^{2}}$ was averaged across dataset versions NA20, NA50, and NA70 and this overall estimate of marker imputation accuracy is referred to as $\overset{–}{\overset{–}{R_{m}^{2}}}$.The median $\overset{–}{\overset{–}{R_{m}^{2}}}$ for each value of MAF rounded to the nearest tenth was calculated. The relationship between the median $\overset{–}{\overset{–}{R_{m}^{2}}}$ and the MAF value was then plotted to characterize the relationship.

The impact of the number of nonmissing data points at a marker on the marker’s imputation accuracy was assessed for each dataset-imputation method combination using data from all 10 replicates and versions NA20, NA50 and NA70 combined. For each marker, the number of nonmissing data points was rounded to the nearest factor of 5, and for each value the median $R_{m}^{2}$was calculated.

To determine the impact of the LD level with other markers on the imputation accuracy, markers were first classified as markers in low LD with all other markers or markers in at least moderate LD with at least one other marker. Markers whose highest r^2^ statistic was less than 0.5 were considered to be in low LD with all other markers. A marker that had at least one r^2^ statistic greater than or equal to 0.5 was considered to be in at least moderate LD with at least one other marker. The median $\overset{–}{\overset{–}{R_{m}^{2}}}$ of markers in low LD and of markers in at least moderate LD with at least one other marker was calculated. The ratio of $\overset{–}{\overset{–}{R_{m}^{2}}}$ for markers in low LD to the $\overset{–}{\overset{–}{R_{m}^{2}}}$ for markers in at least moderate LD was then examined.

To assess the effect of the genetic distance between an individual and its closest relative on the individual genotype imputation accuracy, the Euclidian distance was calculated for each pair of individuals and the $R_{i}^{2}$ of each dataset was measured for each simulated dataset and imputation method combination. The mean $R_{i}^{2}$ values across all replicates, $\overset{–}{R_{l}^{2}}$, were averaged across versions NA20, NA50, and NA70 of a given dataset-imputation method combination to calculate an overall mean $R_{i}^{2}$ for each individual which is referred to as $\overset{–}{\overset{–}{R_{l}^{2}}}$. The Euclidian distance between each individual and its closest relative, rounded to the nearest whole number was plotted against the median $\overset{–}{\overset{–}{R_{l}^{2}}}$ to examine the relationship.

The relationship between PEV for the genetic values and the $\overset{–}{\overset{–}{R_{l}^{2}}}$ was also examined. An individual’s PEV is a measure of genetic connectedness to the other individuals (Kennedy and Trus 1993) where an individual’s connectedness is determined by the number and strength of the genetic relationships between that individual and the other individuals in the dataset. For example, a low PEV indicates high connectedness and high degree of genetic relationship. To measure an overall PEV value for each individual, a vector of PEVs was calculated for each marker using the mixed model equations (Searle *et al.* 1992) implemented in the R package “rrBLUP” (Endelman 2011). The genetic and error variance components were estimated using restricted maximum likelihood, and the genomic relationship matrix, excluding the response variable marker, was used as the covariance matrix between genotypes. The sum of the PEV vectors across all markers was used as the overall PEV vector. Because PEV is a reflection of the number and strength of the genetic relationships between individuals, it is expected to be a useful indicator for how well and individual’s missing data can be imputed using all other individuals as a reference.

### GS accuracy calculation

All 10 simulations of missing data versions NA20, NA50, and NA70 of the WW, SW, DTM, NAB, and SRRW marker sets were imputed with each of the imputation methods: MNI, kNNI, SVDI, EMI, and RFI. Then, each of the 10 replicates of the marker set-imputation method combinations was used to calculate the 10-fold cross validation GS accuracy for both Ridge-Regression (Whittaker *et al.* 2000) and Bayesian LASSO (de los Campos *et al.* 2009), see File S1. GS accuracies are computed as the Pearson’s correlation between the phenotype estimated breeding values and the genomic-estimated breeding values. The mean accuracy for each marker set-imputation method-prediction model combination was computed. GS accuracies were also computed using version NA0 of the WW, SW, DTM, and NAB genotypic data.

## Results

### LD between markers

For each dataset, the LD between marker pairs was quantified using the r^2^ statistic. Markers that had at least one other marker associated with r^2^≥ 0.5 were considered to be in at least moderate LD with at least one other marker. In the WW, SW, DTM, and NAB datasets, 62%, 74%, 12%, and 69% of the markers had at least one other marker in at least moderate LD, respectively. Comparatively, LD between markers was high in the SW, NAB, and WW datasets and much lower in the DTM dataset.

### Imputation accuracy

**Figure 1:**
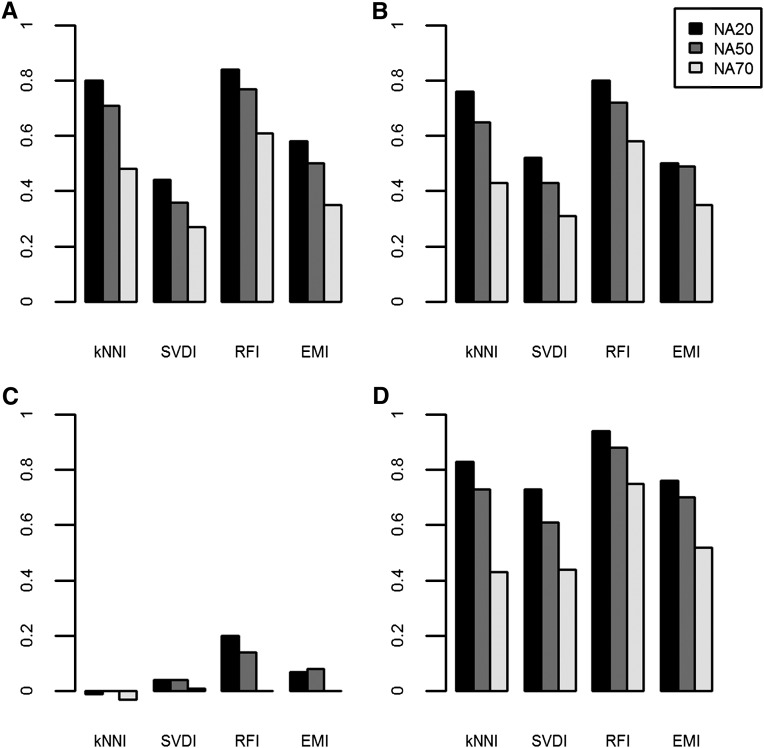
Median $\overset{–}{R_{m}^{2}}$of each imputation method across all datasets. (A) WW; (B) SW; (C) DTM; and (D) NAB. For each population median $\overset{–}{R_{m}^{2}}$ obtained using kNNI, SVDI, RFI, and EMI are shown for the three dataset versions: NA20 (black), NA50 (gray), and NA70 (white), which contain up to 20%, 50%, and 70% missing values per marker, respectively.

The imputation accuracy reported as the median $\overset{–}{R_{m}^{2}}$ is shown in Figure 1 for kNNI, SVDI, RFI, and EMI. For all dataset-imputation method combinations, $\overset{–}{R_{m}^{2}}$ values were non-normal, and there were many extreme values. The median $\overset{–}{R_{m}^{2}}$ values and the equivalent percent correct values are listed in Table 2. The population with the highest median $\overset{–}{R_{m}^{2}}$ for each of the levels of missing data were the NAB population, whereas the lowest imputation accuracies were observed with the DTM population. As expected, median $\overset{–}{R_{m}^{2}}$ values always decreased as the level of missing data increased. RFI always produced the highest accuracies; kNNI generally produced the second highest accuracies, followed by EMI and SVDI. The rankings were slightly different for the DTM dataset, where RFI was most accurate followed by EMI, SVDI, and kNNI. The rankings of the methods for each dataset according to the median percent correct are the same as those according to the median $\overset{–}{R_{m}^{2}}$; however, the median percent correct values could not be compared across datasets because percent correct values are influenced by the MAF which differs among datasets.

**Table 2:** Median $\overset{–}{R_{m}^{2}}$ and median percent correct for each imputation method and across all datasets

		Imputation Method			
Dataset	Version*^a^*	kNNI	SVDI	RFI	EMI
WW	NA20	0.8 / 97	0.44 / 93	0.84 / 98	0.58 / 95
	NA50	0.71 / 96	0.36 / 92	0.77 / 97	0.5 / 93
	NA70	0.48 / 94	0.27 / 89	0.61 / 95	0.35 / 91
	Mean	0.66 / 96	0.36 / 91	0.74 / 97	0.48 / 93
SW	NA20	0.76 / 96	0.52 / 93	0.8 / 97	0.5 / 93
	NA50	0.65 / 95	0.43 / 93	0.72 / 96	0.49 / 93
	NA70	0.43 / 93	0.31 / 91	0.58 / 94	0.35 / 91
	Mean	0.61 / 95	0.42 / 92	0.7 / 96	0.45 / 92
DTM	NA20	−0.01 / 82	0.04 / 83	0.2 / 88	0.07 / 85
	NA50	0 / 82	0.04 / 83	0.14 / 87	0.08 / 84
	NA70	−0.03 / 82	0.01 / 83	0 / 84	0 / 83
	Mean	−0.01 / 82	0.03 / 83	0.11 / 86	0.05 / 84
NAB	NA20	0.83 / 99	0.73 / 98	0.94 / 100	0.76 / 98
	NA50	0.73 / 99	0.61 / 98	0.88 / 99	0.7 / 98
	NA70	0.43 / 97	0.44 / 97	0.75 / 99	0.52 / 97
	Mean	0.66 / 98	0.59 / 98	0.85 / 99	0.66 / 98

Median $\overset{–}{R_{m}^{2}}$ and median percent correct are separated by a backslash (/). kNNI, k-nearest neighbors imputation; SVDI, singular value decomposition imputation; EMI, expectation maximization imputation; RFI, random forest regression imputation; WW, Cornell winter wheat; SW, CIMMYT elite spring wheat; DTM, CIMMYT drought-tolerant maize; NAB, North American barley.

a NA20: up to 20% missing data per marker, NA50: up to 50% missing data per marker, NA70: up to 70% missing data per marker.

### Computational time

Large differences in the computational requirements for the imputation methods were observed (Table 3). kNNI, SVDI, and EMI required relatively little computation time on average, while RFI required at least 95x, 760x, and 65x more computation time than kNNI, SVDI, and EMI, respectively. For SVDI and kNNI, the computation time required for determining optimal k values was not included in the estimates of the average computational time because the computation time for optimal k estimation depends on the method used for estimation. The 10-fold cross validation approach that we used to estimate optimal k values for SVDI and kNNI requires approximately 50 runs of the SVDI and kNNI respectively. If the time required to estimate optimal k values for SVDI and kNNI was included in the total computational time, EMI would be the fastest of the four imputation methods.

**Table 3:** CPU minutes required to complete the imputation of one dataset

		Imputation Method			
Dataset	Version*^a^*	kNNI	SVDI	RFI	EMI
WW	NA20	2.5	0.4	364.8	2.2
	NA50	4.7	0.4	411.6	3.1
	NA70	5.6	0.4	280.2	2.7
SW	NA20	5.3	1.5	132.6	5.5
	NA50	9.7	1.5	935.4	9.1
	NA70	11.5	1.5	610.2	7.3
DTM	NA20	1.7	0.2	271.8	0.8
	NA50	3.3	0.2	440.4	0.8
	NA70	4.1	0.2	223.8	1.0
NAB	NA20	24.4	6.0	4084.8	64.6
	NA50	45.1	5.8	4204.2	106.7
	NA70	50.3	5.8	2349	86.2
SRRW	NA20	7.1	0.7	2364.6	3.5
	NA50	14.2	0.6	1618.8	4.8
	NA70	17.1	0.6	1309.2	4.1

CPU, central processing unit; kNNI, k-nearest neighbors imputation; SVDI, singular value decomposition imputation; EMI, expectation maximization imputation; RFI, random forest regression imputation; WW, Cornell winter wheat; SW, CIMMYT elite spring wheat; DTM, CIMMYT drought-tolerant maize; NAB, North American barley; SRRW, CIMMYT stem rust-resistant wheat.

a NA20: up to 20% missing data per marker, NA50: up to 50% missing data per marker, NA70: up to 70% missing data per marker.

### Factors affecting imputation accuracy

#### MAF:

**Figure 2:**
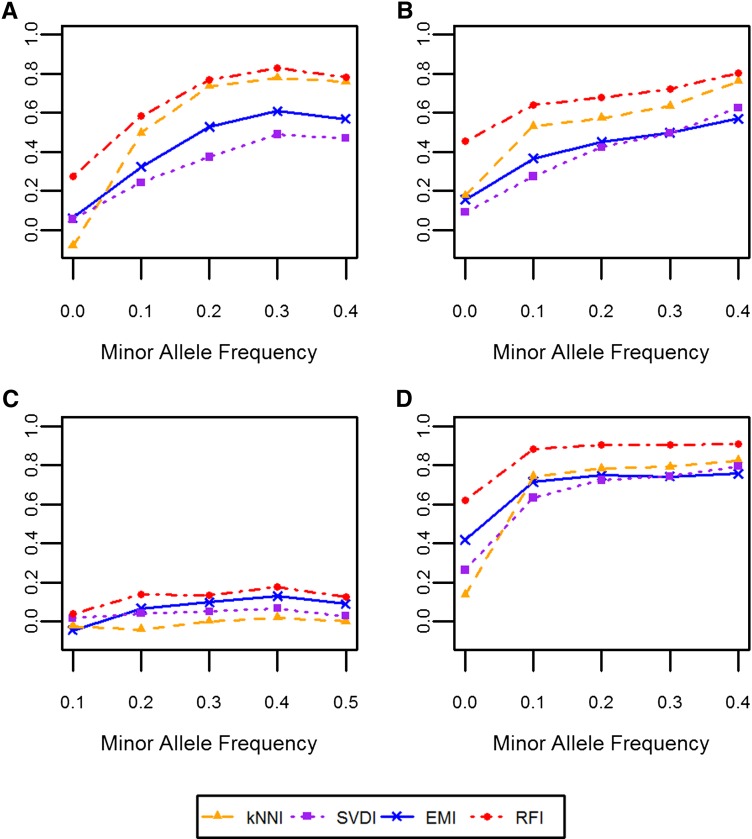
Relationship between the MAF and $\overset{–}{\overset{–}{R_{m}^{2}}}$_._ The median $\overset{–}{\overset{–}{R_{m}^{2}}}$obtained for a given MAF rounded to the nearest tenth is plotted for each dataset: (A) WW; (B) SW; (C) DTM; and (D) NAB. Each color and symbol represents a different imputation method: kNNI, orange triangles; SVDI, purple squares; RFI, red circles; and EMI, blue crosses.

For all datasets, $\overset{–}{\overset{–}{R_{m}^{2}}}$ values for markers with MAF < 0.1 were low compared with that of markers with MAF > 0.1; however, the relationship between MAF and $\overset{–}{\overset{–}{R_{m}^{2}}}$ for markers with MAF > 0.1 was different for each dataset (Figure 2). In general, $\overset{–}{\overset{–}{R_{m}^{2}}}$ increased as MAF increased as long as MAF < 0.4; however, with the NAB dataset (Figure 2D) there was no relationship between MAF and $\overset{–}{\overset{–}{R_{m}^{2}}}$ for MAF > 0.1. Accuracy in terms of percent correct had a strong negative linear relationship with the MAF across all imputation methods and datasets. Markers with lower MAF values tended to have higher percent correct values (data not shown).

#### Number of nonmissing data points:

**Figure 3:**
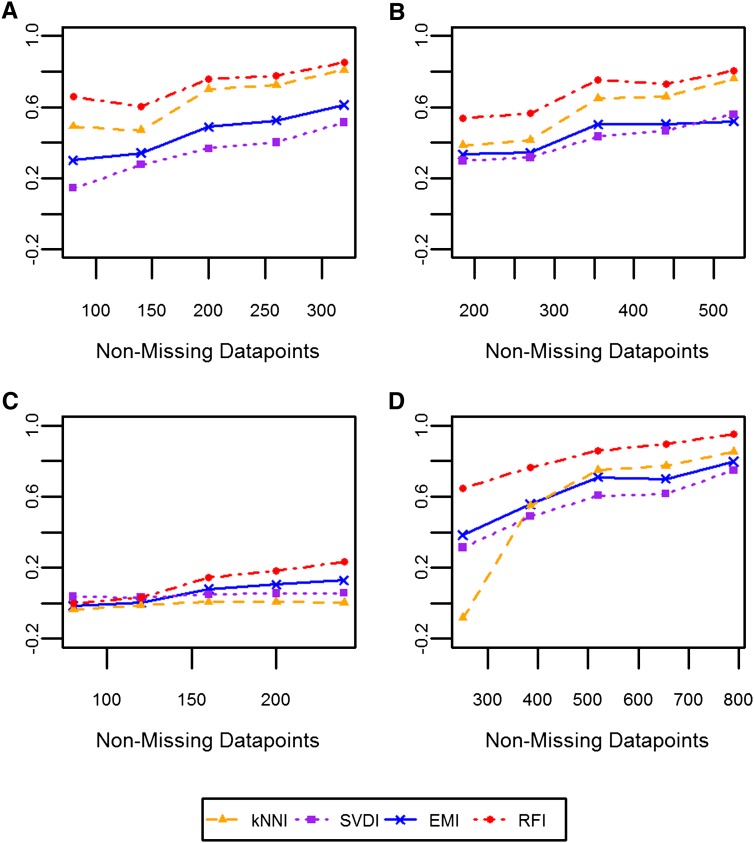
Relationship between the number of nonmissing datapoints and $R_{m}^{2}$. The median $R_{m}^{2}$ obtained for a given number nonmissing data points rounded to the nearest factor of 5, is plotted for each dataset: (A) WW; (B) SW; (C) DTM; and (D) NAB. Each color and symbol represents a different imputation method: Each color and symbol represents a different imputation method: kNNI, orange triangles; SVDI, purple squares; RFI, red circles; and EMI, blue crosses.

With almost all dataset-imputation method combinations, as the number of non-missing data points increased, the $\overset{–}{R_{m}^{2}}$ levels increased in a linear fashion (Figure 3). The strength of this linear relationship was similar for all imputation methods; however, with the DTM dataset, $\overset{–}{R_{m}^{2}}$ for kNNI and SVDI were close to zero regardless of the number of nonmissing data points.

#### LD between markers:

The ratio of the median imputation $\overset{–}{\overset{–}{R_{m}^{2}}}$ for markers with no other markers in moderate LD to the median imputation $\overset{–}{\overset{–}{R_{m}^{2}}}$ for markers with at least one other marker in moderate LD was always less than one (Table 4), indicating that the imputation accuracy for markers without markers in moderate LD was always lower than that for markers that had at least one other marker in moderate LD. Across all datasets, the $\overset{–}{\overset{–}{R_{m}^{2}}}$ ratios for the two classes of markers was much smaller for kNNI compared to the other imputation methods, indicating that the imputation accuracy of kNNI was more strongly influenced by the level of LD between markers compared to the other methods. With the WW, SW, and NAB datasets the $\overset{–}{\overset{–}{R_{m}^{2}}}$ ratios for the two classes of markers was similar for SVDI, RFI, and EMI indicating that the accuracy of these three methods is influenced by the level of LD between markers to a similar degree. However, with the DTM dataset, the $\overset{–}{\overset{–}{R_{m}^{2}}}$ ratio for the two classes of markers was closer to one for SVDI compared to the other methods, indicating that for this dataset, the accuracy with SVDI was less affected by the LD between markers, compared to the other methods.

**Table 4:** Ratios of median $\overset{–}{\overset{–}{R_{m}^{2}}}$of markers having no markers in moderate linkage disequilibrium (LD) to that of markers with at least one other marker in moderate LD

		Imputation Method			
Dataset	kNNI	SVDI	RFI	EMI
WW	0.16 (0.13/0.8)	0.36 (0.17/0.47)	0.49 (0.41/0.84)	0.39 (0.23/0.59)
SW	0.14 (0.1/0.7)	0.47 (0.23/0.49)	0.62 (0.47/0.76)	0.58 (0.29/0.5)
DTM	−0.18 (−0.03/0.17)	0.33 (0.02/0.06)	0.18 (0.09/0.5)	0.14 (0.03/0.22)
NAB	0.31 (0.24/0.78)	0.59 (0.40/0.68)	0.74 (0.67/0.9)	0.63 (0.46/0.73)
Mean	0.11	0.44	0.51	0.44

Reduced ratios are reported followed by the values used to compute the reduced ratios in parenthesis. At least moderate LD was defined as r^2^ statistic ≥ 0.5. kNNI, k-nearest neighbors imputation; SVDI, singular value decomposition imputation; EMI, expectation maximization imputation; RFI, random forest regression imputation; WW, Cornell winter wheat; SW, CIMMYT elite spring wheat; DTM, CIMMYT drought-tolerant maize; NAB, North American barley.

#### Distance from the closest relative and PEV:

**Figure 4:**
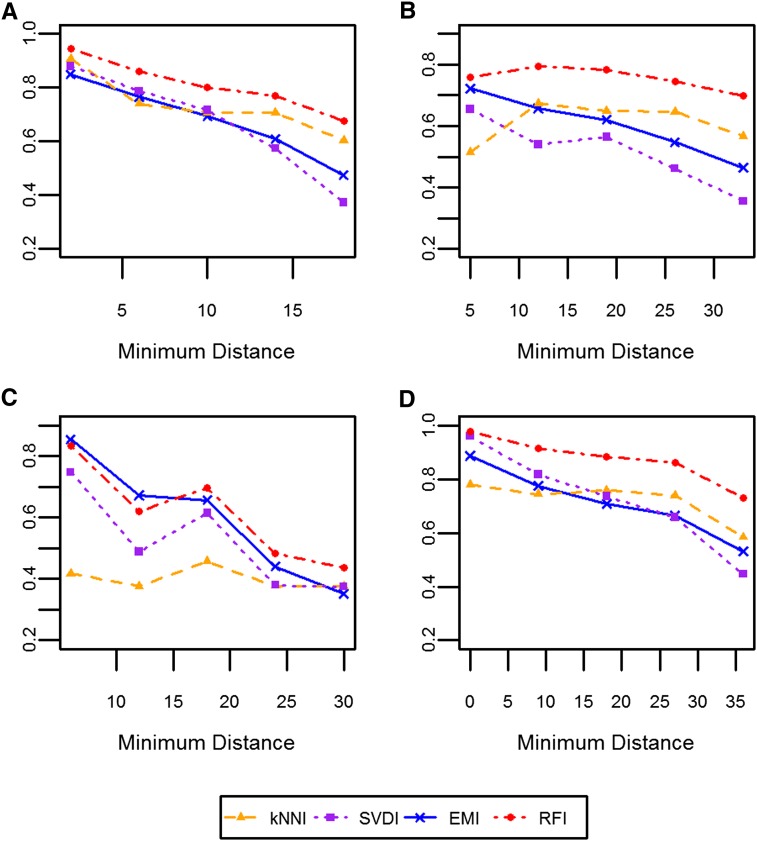
Relationship between the distance from the closest relative and $\overset{–}{\overset{–}{R_{l}^{2}}}$_._ The median $\overset{–}{\overset{–}{R_{l}^{2}}}$ obtained for a given Euclidean distance between an individual and its closest relative rounded to the nearest whole number is plotted for each dataset: (A) WW; (B) SW; (C) DTM; and (D) NAB. Each color and symbol represents a different imputation method: kNNI, orange triangles; SVDI, purple squares; RFI, red circles; and EMI, blue crosses.

Regardless of the dataset or the imputation method, the smaller the distance between an individual and its closest relative, the higher the $\overset{–}{\overset{–}{R_{l}^{2}}}$ (Figure 4). One exception was observed with the DTM dataset, where for kNNI there was no relationship between the distance between an individual and its closest relative and $\overset{–}{\overset{–}{R_{l}^{2}}}$. We observed very similar trends between $\overset{–}{\overset{–}{R_{l}^{2}}}$ and the overall PEV (Figure S2). As an individual’s PEV increased, indicating a decrease in the strength and number of genetic relationships between that individual and all other individuals, its $\overset{–}{\overset{–}{R_{l}^{2}}}$ decreased in all cases except when the DTM dataset was imputed with kNNI.

### Effect of imputation method on GS accuracy

**Figure 5:**
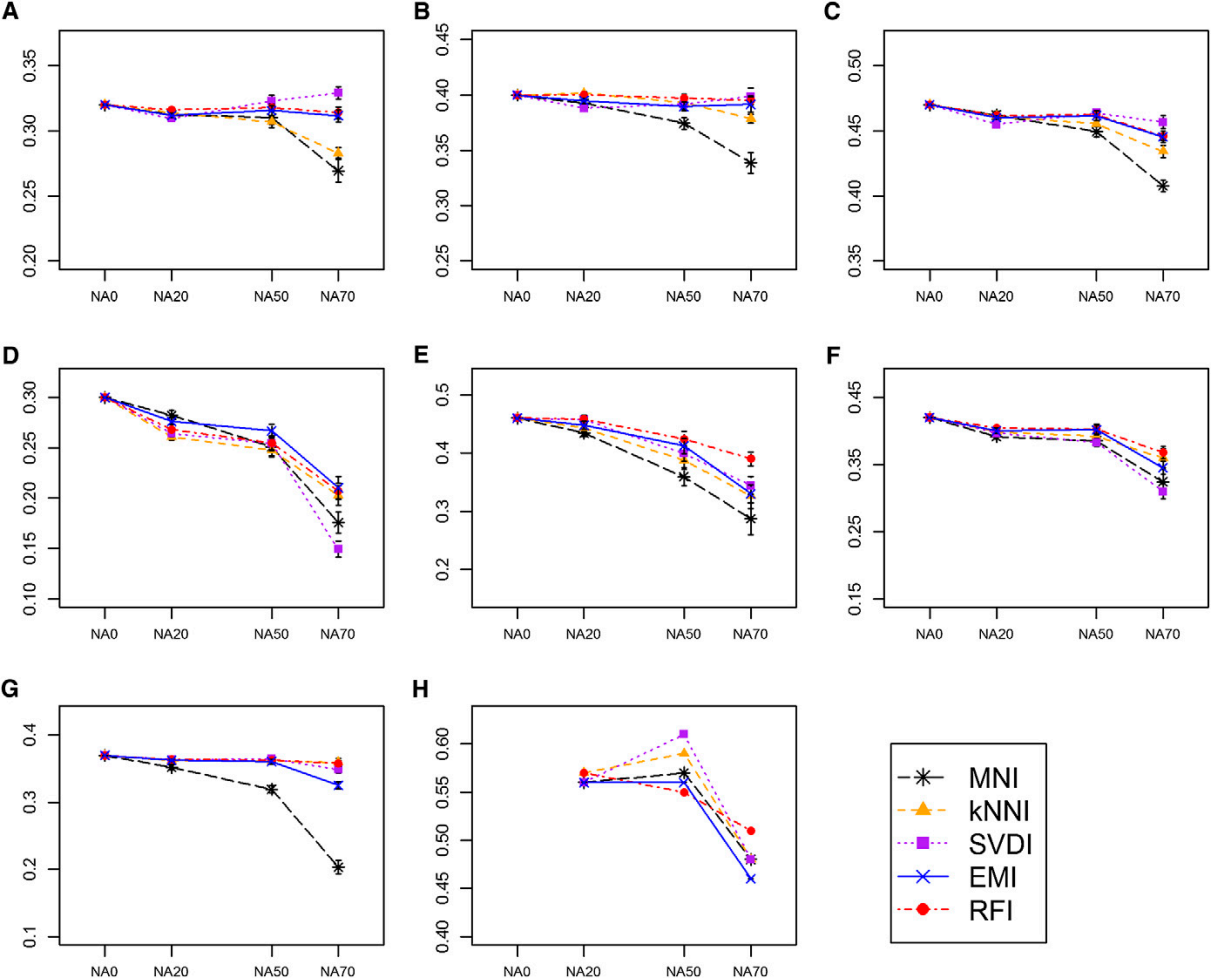
GS accuracy obtained using ridge regression after imputation. Mean GS accuracies obtained using the dataset versions NA0, NA20, NA50, having up to 0%, 20%, 50%, and 70% missing data per marker, respectively, imputed with either MNI (black stars), kNNI (orange triangles), SVDI (purple squares), EMI (blue crosses) and RFI (red circles) are shown for (A) WW-yield, (B) WW-height, (C) WW-protein, (D) WW-days to heading, (E) DTM, (F) SW, (G) NAB, and (H) SRRW datasets. Each plot has a different y-axis range. Error bars depict SE.

**Figure 6:**
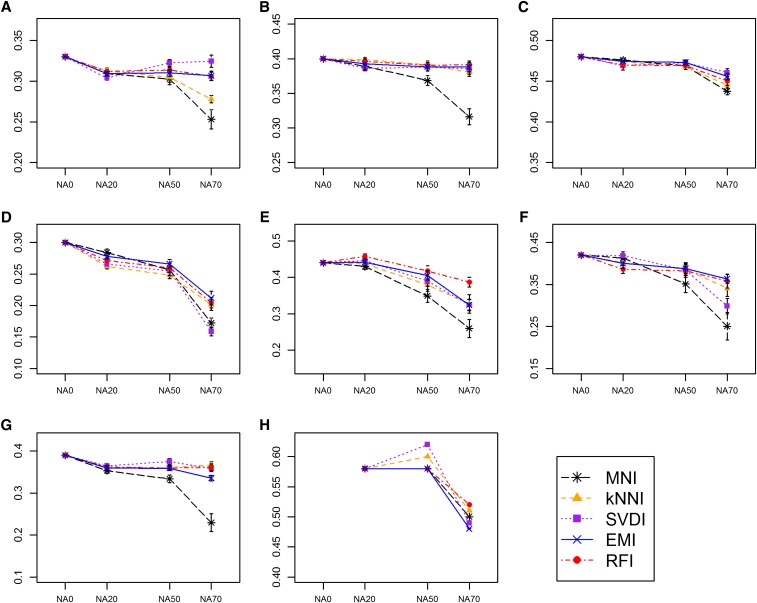
GS accuracy obtained using Bayesian least absolute shrinkage and selection operator after imputation. Mean GS accuracies obtained using the dataset versions NA0, NA20, and NA50, having up to 0%, 20%, 50%, and 70% missing data per marker respectively, imputed with either MNI (black stars), kNNI (orange triangles), SVDI (purple squares), EMI (blue crosses) and RFI (red circles) are shown for (A) WW-yield, (B) WW-height, (C) WW-protein, (D) WW-days to heading, (E) DTM, (F) SW, (G) NAB, and (H) SRRW datasets. Each plot has a different y-axis range. Error bars depict SE.

In nearly all cases, GS accuracies did not differ greatly from one imputation method to another, with the exception of MNI, which sometimes led to much lower accuracies compared to all other methods when the NA70 dataset version was used (Figure 5 and 6). Overall, GS accuracies were least affected by the imputation method for dataset version NA20, and most affected by the imputation method for dataset version NA70. The relative performance of each method in terms of GS accuracy after imputation depended on the dataset, and dataset version; however, RFI consistently performed well across all datasets. For the WW datasets, the relative performance of the imputation methods in terms of GS accuracy was inconsistent across the four traits tested.

For, a given dataset and dataset version, the rank of each method based on $\overset{–}{R_{m}^{2}}$, was not consistent with the rank based on GS accuracy using RR or BL post-imputation. The rank of the imputation methods, however, was consistent between the two different GS models. We also found that including rather than removing ‘sparse’ markers, those with large amounts of missing data, nearly always led to higher GS accuracies (methods and results described in File S1, Table S2, and Figure S3), especially when RFI, kNNI, or EMI were the imputation methods used (Figure S4).

## Discussion

### Imputation accuracy

This study found that map-independent imputation methods other than MNI can be surprisingly accurate, especially when LD between markers is high and the genotyped individuals are related. RFI was the most promising method overall because of its consistently high performance in terms of imputation accuracy and subsequent GS accuracy; however, it was the most computationally intensive method evaluated. kNNI, although less accurate than RFI, may be a good alternative to RFI if there are computational limitations to completing the imputation. It is likely that RFI and kNNI produced comparable levels of accuracy because both use a similar model free approach for imputation that involves weighting a selected set of k important variables according to a distance metric (Lin and Jeon 2006). The weighted average of these variables is the predicted value of the variable of interest. For kNNI the distance metric was the Euclidean distance and k was a fixed number across all variables. For RFI, the k important variables and their weights are determined by the splitting scheme of the tree that is determined using the response variable. The increased accuracy but greater computational burden of the RFI method compared with kNNI is due to its adaptive weighting of variables that takes into account the response variable.

A possible reason that EMI and SVDI were less accurate than RFI and kNNI is that the genotypic datasets that we used may have violated multivariate normality, an underlying assumption for EMI and SVDI. Alternatively, EMI and SVDI may not have been as effective at ignoring uninformative predictors. If true, linear regression based imputation methods involving variable selection could be as accurate as kNNI or RFI. However, due to multicollinearity, attempts to test imputation based on subset selection methods such as stepwise regression were not successful. Regression imputation using variable selection methods which can cope with multicollinearity, such as the least absolute shrinkage and selection operator (Tibshirani 1996), would be interesting to test in future studies.

EMI performed consistently better than SVDI which is likely because EMI incorporates all the marker data as predictors whereas SVDI first used a data reduction step, potentially eliminating useful information. SVDI may have outperformed EMI if the datasets had a greater rate of genotyping error because it is expected to better cope with noisy data (Troyanskaya *et al.* 2001).

For all methods, average median imputation accuracies on an individual genotype basis $\overset{–}{\overset{–}{R_{l}^{2}}}$ were not always homogenous across population sub-groups as illustrated in Figure S5, which shows individuals plotted according to the first two principal components of their marker genotypes and color coded according to their imputation accuracy. With the DTM and WW datasets, small subgroups of individuals that clustered together according to the first two principal components of marker genotypes tended to have similar ranges of accuracy. However, with the SW and NAB datasets $\overset{–}{\overset{–}{R_{l}^{2}}}$ was relatively homogenous across population subgroups. An association between $\overset{–}{\overset{–}{R_{l}^{2}}}$ and population subgroup is undesirable because it may create or worsen an association between GS accuracy and population sub-group. Using large datasets with minimal population structure for imputation and GS is advocated to avoid heterogeneity of imputation and GS accuracies across subgroups of individuals.

Population structure may also lead to increased imputation accuracy for markers with high levels of population subdivision (Iwata and Jannink 2010) because an individual’s allelic state can be predicted largely based population sub-group alone. Accuracy levels for datasets with many markers highly subdivided by population may be high largely because of structure; we therefore calculated $\overset{–}{\overset{–}{R_{m}^{2}}}$ excluding markers with high levels of population subdivision as indicated by their Fst values, where high Fst indicates high population subdivision (for methods, see File S1). For markers with MAF > 0.1, on average, $\overset{–}{\overset{–}{R_{m}^{2}}}$ excluding markers with the 25% highest Fst values were 0.9, 1.17, 1.02, and 0.9 times those of overall $\overset{–}{\overset{–}{R_{m}^{2}}}$ for the WW, SW, DTM, and NAB datasets, respectively. Thus, for the WW and NAB datasets, the high imputation accuracies we observed may have been in small part due to population structure.

Comparing our imputation accuracy results with those of other studies is difficult because each study uses different populations of different sizes, levels of missing data, MAF distributions, and levels of LD between markers. In addition, accuracy reported as percent correct cannot be compared across datasets with different MAF distributions. Nevertheless, we assume that map-dependent imputation methods would outperform the map-independent methods that we evaluated (given the availability of an accurate genetic or physical map) because physically linked markers are used to predict missing values. These physically linked markers should be more reliable predictors compared to markers that are in LD but may not be physically linked. As genetic and physical maps develop for wheat and barley the assumption that map-dependent methods would outperform the map-independent methods can be tested.

### Factors affecting imputation accuracy

Markers with very low MAF had low $\overset{–}{\overset{–}{R_{m}^{2}}}$ values. There are two possible explanations for this observation. First, because of the way $R_{m}^{2}$ is calculated, a single imputation error has a much larger negative impact on the $R_{m}^{2}$ for markers with lower MAF values (Figure S6). Thus, it is harder to achieve high $R_{m}^{2}$ for markers with a low MAF. Second, individuals with the minor allele at a given marker are not well represented, making their marker genotype more difficult to predict. A similar relationship between MAF and $R_{m}^{2}$ was also found by studies by Iwata and Jannink (2010) and Li *et al.* (2011), which used map-dependent imputation methods. Unlike $R_{m}^{2}$, imputation accuracy in terms of percent correct had a negative linear relationship with MAF (data not shown), this is because markers with lower MAF can always be imputed with a reasonably high percent correct based on the marker mean alone. Other studies of map-dependent imputation methods report a negative relationship between MAF and percent correct (Pei *et al.* 2008; Hickey *et al.* 2012).

The number of nonmissing data points, analogous to reference panel size in other studies was found to positively impact the $R_{m}^{2}$. This finding is consistent with other studies in which researchers tested the effect of reference panel size on the imputation accuracy using map-dependent methods (Pei *et al.* 2008; Druet *et al.* 2010; Li *et al.* 2010). For RFI, EMI, and SVDI, which involve a model training step, fewer missing data points means that more individuals are available for model training. With kNNI, a smaller number of nonmissing data points at a given marker leads to a more accurate estimate of its distance from all other markers. However, with the DTM set there was no trend between accuracy and the number of nonmissing data points with kNNI. This was because accuracy with kNNI for this dataset was very low overall.

The presence of one or more markers in moderate LD (r^2^ statistic ≥ 0.5) was a more important factor for kNNI compared with RFI, EMI, and SVDI because kNNI bases its predictions on a fixed number of close markers, whereas RFI, EMI, and SVDI use information from all markers in the dataset to generate predicted values for the missing data points. The LD between markers on a whole dataset basis also appeared to be an important factor affecting the $\overset{–}{\overset{–}{R_{m}^{2}}}$ of all methods because accuracies with the DTM dataset, which had low levels of LD between markers overall, were much lower than accuracies with the WW, SW, and NAB datasets. Other publications that have evaluated the effect of LD on imputation accuracy for map-dependent methods have found similar trends (Pei *et al.* 2008; Hickey *et al.* 2012).

We found that imputation accuracy on an individual genotype level was negatively correlated with the distance from the closest relative in the dataset, and the PEV, which is an indication of the relationship between an individual and other genotypes. A similar relationship between imputation accuracy and relationship has been found by other studies of map-dependent imputation methods (Druet *et al.* 2010; Zhang and Druet 2010; Hickey *et al.* 2012). It is clear that to ensure effective imputation, the dataset to be imputed should contain related individuals. If the dataset is suited for GS, it is likely that the individuals are already related. However, to increase the chances that an individual will have close relatives in the dataset, all available genotypic data for the germplasm pool of interest should be combined before imputation.

### GS accuracy

The GS accuracies that we observed may be sufficiently high to lead to increased rates of genetic gain compared to phenotypic selection (PS), depending on the accuracy of PS and the selection cycle duration of both PS and GS. It is important to note that all GS accuracies reported for a given dataset are global estimates across all potential subpopulations. Based on other studies evaluating GS accuracies within and across subpopulations (Zhao *et al.* 2011, Heslot *et al.* 2012, Windhausen *et al.* 2012), this global accuracy estimate may be greater than the accuracy measured within individual subpopulations.

### Effect of imputation method on the GS accuracy

Improved accuracy of GS after application of map independent imputation methods was another important finding of this study. Based on our results, unordered markers with missing data can be included in the dataset to improve accuracy through imputation with RFI, kNNI, EMI, or even SVDI rather than MNI. However, for datasets with low levels of missing data (up to 20% per marker), imputing with MNI is sufficient. Although our results do not support removing markers with high levels of missing data prior to GS, in many datasets markers with low levels of missing data may be sufficient to saturate the genome. With the datasets used in this study, the average number of markers with up to 20% and 50% missing data were 18 to 37 and 99 to 186 respectively, and these reduced marker sets were not sufficient to saturate the genome. Thus, including markers with larger amounts of missing data led to improved GS accuracies. Interestingly, a low median $\overset{–}{R_{m}^{2}}$ was not reflective of the merit of imputation prior to GS. The median $\overset{–}{R_{m}^{2}}$ for the datasets with up to 70% missing data per marker were the lowest of all the missing data levels; however we saw the greatest gain in GS accuracy from kNNI, SVDI, EMI, or RFI relative to MNI with this level of missing data. This was especially apparent for the DTM dataset, which had a median $\overset{–}{R_{m}^{2}}$ near zero for most methods when there was up to 70% missing data per marker. However, RFI on this dataset produced GS model accuracies 1.3 times greater than those achieved when MNI was used before GS. Surprisingly, the most accurate imputation method was not always the method that gave the greatest GS accuracy. This may be caused by nonrandom imputation errors. If some imputation errors are similar for related individuals, these nonrandom errors may able to capture some genetic relationships in the GS model. The idea that the imputation errors may capture some genetic relationships was suggested by a study by Weigel *et al.* (2010).

This study has important implications for species that lack a reference genome, complete reference map, and predesigned high-throughput genotyping platforms. First, unordered markers can be imputed with high levels of accuracy, and even greater accuracies may result if additional reference genotypes can be added to the dataset prior to imputation. Based on the results of this study, if a large number of marker genotypes are produced (so that markers are in LD with each other), and the population contains individuals with some genetic relationship, missing data can be imputed with reasonable accuracy even if the level of missing data are high; up to 70%. Future work to improve upon and reduce the computational burden of the most promising methods in this study, RFI and kNNI, would be especially useful if these methods are to be used widely. The second implication of this study is that a large proportion of missing data in dense marker sets is not a major concern for GS. As long as the marker density is sufficiently high, the accuracy does not appear to be strongly negatively affected. In cases where missing data does negatively impact the GS accuracy imputation using a method other than MNI before GS model training and validation can help improve the accuracy. Overall, map-independent imputation shows promise for the feasibility of applying GS, enabled by emergent sequence-based genotyping technologies, to almost any species regardless of the availability of pre-existing genotyping resources.

## Supplementary Material

Supporting Information
