# Supplementary material for: Imputation of Unordered Markers and the Impact on Genomic Selection Accuracy
Source: G3 (Bethesda). 2013 Mar 1;3(3):427–39. doi: 10.1534/g3.112.005363 (PMC3583451; doi:10.1534/g3.112.005363)
Supplement: Supporting Information [file supp_3_3_427__index.html]

Supporting Information 

# Imputation of Unordered Markers and the Impact on Genomic Selection Accuracy

## Supporting Information for Rutkoski *et al.*, 2013

**Files in this Data Supplement:**

- Supporting Information - Figures S1-S6, Tables S1 and S2, and Files S1-S6 (PDF, 2.8 MB)
- Figure S1 - Illustration of example dataset versions NA20, NA50, and NA70 (PDF, 189 KB)
- Figure S2 - Relationship between the overall expected prediction error variance (PEV) and (R ̿2i) (PDF, 139 KB)
- Figure S3 - Illustration of the construction of marker sets used to determine the effect of excluding sparse marker data on the genomic selection accuracy (PDF, 176 KB)
- Figure S4 - The effect of excluding sparse marker data on the genomic selection accuracy (PDF, 163 KB)
- Figure S5 - Heterogeneity of accuracies across population sub-groups (PDF, 902 KB)
- Figure S6 - The relationship between imputation accuracy measured as R2m and measured as percent correct for different minor allele frequencies (PDF, 118 KB)
- Table S1 - Optimal k values for KNNIa and SVDIb used across all replicates (PDF, 82 KB)
- Table S2 - Description of datasets used to test the effect of excluding sparse marker data on the genomic selection accuracy (PDF, 70 KB)
- File S1 - Supporting Text (PDF, 219 KB)
- File S2 - Cornell winter wheat dataset (.zip, 178 KB)
- File S3 - Stem rust resistant wheat dataset version NA20 (.zip, 243 KB)
- File S4 - Stem rust resistant wheat dataset version NA50 (.zip, 276 KB)
- File S5 - Stem rust resistant wheat dataset version NA70 (.zip, 258 KB)
- File S6 - R code and workspace for example imputation (.zip, 273 KB)
