# Supplementary material for: Imputation of Unordered Markers and the Impact on Genomic Selection Accuracy
Source: G3 (Bethesda). 2013 Mar 1;3(3):427–39. doi: 10.1534/g3.112.005363 (PMC3583451; doi:10.1534/g3.112.005363)
Supplement: Supporting Information [file supp_3.3.427_005363SI.pdf]

G3 · Genes | Genomes | Genetics

IMPUTATION OF UNORDERED MARKERS AND THE IMPACT ON GENOMIC SELECTION ACCURACY

Jessica E. Rutkoski*, Jesse Poland§, Jean-Luc Jannink§§, Mark E. Sorrells*

*Dept. of Plant Breeding and Genetics; Cornell University; 240 Emerson Hall, Ithaca NY, 14853.
§United States Department of Agriculture; Agricultural Research Service (USDA-ARS) and Department of Agronomy; Kansas State University (KSU); 4011 Throckmorton Hall, Manhattan KS, 66506.
§USDA-ARS and Department of Plant Breeding and Genetics, Cornell University, 240 Emerson Hall, Ithaca, NY 14853.

Corresponding author: Mark E. Sorrells Department of Plant Breeding & Genetics, 240 Emerson Hall, Cornell, Ithaca, NY 14853-1902, 607-255-2180, 607-255-6683, mes12@cornell.edu

DOI: 10.1534/g3.112.005363

Version NA0

	m1	m2	m3	m4	m5	m6	m7	m8	m9	m10	m11	m12	m13	m14	m15	m16	m17	m18	m19	m20
g1	1	0	1	-1	-1	1	-1	1	1	1	0	-1	-1	0	1	1	-1	-1	-1	0
g2	-1	0	-1	0	1	0	1	-1	0	0	-1	0	0	1	0	0	1	-1	1	-1
g3	1	-1	1	0	0	0	-1	1	1	1	1	1	0	1	0	1	1	1	-1	-1
g4	-1	1	0	-1	0	1	0	0	-1	1	1	-1	0	-1	0	0	1	1	0	-1
g5	1	1	-1	0	-1	-1	0	1	-1	-1	-1	-1	0	0	1	-1	-1	0	-1	0
g6	1	0	0	-1	-1	0	1	-1	0	0	-1	1	0	-1	1	1	-1	1	-1	-1
g7	0	0	-1	-1	1	1	-1	1	-1	0	-1	1	1	1	0	1	-1	1	-1	0
g8	1	0	0	-1	-1	1	-1	-1	-1	-1	0	-1	-1	-1	-1	1	-1	-1	0	-1
g9	0	-1	-1	1	-1	-1	0	1	0	1	1	-1	1	-1	0	0	0	0	0	0
g10	1	1	0	0	1	-1	1	0	0	0	1	0	1	1	0	0	0	-1	1	1
g11	0	0	1	1	-1	1	-1	0	0	1	1	1	-1	1	1	0	0	-1	0	0
g12	-1	1	1	-1	0	0	0	-1	-1	1	1	1	0	0	1	-1	0	0	-1	1
g13	1	-1	0	-1	0	0	1	-1	1	0	0	-1	0	1	0	-1	-1	0	1	0
g14	-1	-1	-1	1	0	1	-1	0	1	0	1	-1	-1	1	1	-1	0	1	-1	-1
g15	0	-1	0	-1	1	0	-1	1	0	0	1	-1	1	0	0	1	1	0	-1	0

Version NA20

	m1	m2	m3	m4	m5	m6	m7	m8	m9	m10	m11	m12	m13	m14	m15	m16	m17	m18	m19	m20
g1	1	0	1	-1	-1	1	-1	1	1	1	0	-1	-1	0	1	1	-1	-1	-1	0
g2	-1	NA	-1	0	1	0	1	-1	0	0	-1	0	0	NA	-1	0	1	NA	-1	-1
g3	1	-1	NA	-1	NA	-1	-1	1	1	1	1	1	0	1	0	NA	-1	1	-1	-1
g4	NA	1	0	-1	0	NA	-1	0	-1	NA	-1	-1	0	-1	0	0	1	1	0	-1
g5	1	1	-1	0	-1	-1	0	NA	-1	-1	-1	-1	0	0	1	-1	NA	-1	-1	0
g6	1	0	0	-1	NA	-1	NA	-1	0	0	-1	1	NA	-1	1	1	-1	1	-1	-1
g7	0	0	-1	-1	1	1	-1	1	-1	0	-1	NA	-1	1	0	1	-1	1	-1	0
g8	1	0	NA	-1	-1	1	-1	-1	-1	NA	-1	-1	-1	-1	NA	NA	-1	-1	0	NA
g9	NA	-1	-1	1	-1	NA	-1	1	0	1	1	-1	1	-1	0	0	0	NA	-1	0
g10	1	1	0	0	1	-1	NA	-1	NA	-1	1	0	1	1	0	0	0	-1	1	1
g11	0	0	1	1	-1	NA	-1	0	0	1	1	1	-1	1	1	0	NA	-1	0	0
g12	-1	1	1	-1	0	0	NA	-1	-1	1	NA	-1	0	NA	-1	-1	0	0	-1	NA
g13	1	-1	0	-1	0	0	1	-1	1	NA	-1	-1	0	1	0	-1	NA	-1	1	0
g14	-1	-1	NA	-1	0	1	-1	0	1	0	1	-1	NA	-1	1	-1	0	1	-1	-1
g15	0	-1	0	-1	1	0	-1	1	0	0	NA	-1	1	0	0	NA	-1	0	-1	0

Version NA50

	m1	m2	m3	m4	m5	m6	m7	m8	m9	m10	m11	m12	m13	m14	m15	m16	m17	m18	m19	m20
g1	NA	0	NA	NA	-1	1	-1	NA	1	1	NA	NA	-1	0	NA	1	NA	-1	NA	NA
g2	NA	0	-1	0	NA	0	NA	-1	NA	0	-1	0	0	NA	0	NA	1	-1	NA	NA
g3	1	-1	1	0	0	NA	-1	1	1	NA	1	NA	NA	1	0	1	NA	NA	-1	NA
g4	-1	1	NA	NA	0	1	0	0	-1	1	1	-1	0	-1	0	0	1	1	0	-1
g5	NA	NA	-1	0	NA	-1	NA	1	NA	-1	-1	-1	NA	NA	1	NA	-1	0	NA	0
g6	1	0	0	-1	-1	0	1	NA	NA	NA	-1	1	0	-1	1	1	NA	NA	NA	-1
g7	NA	NA	NA	NA	1	1	-1	1	NA	0	-1	1	1	NA	0	1	NA	1	-1	NA
g8	1	0	0	-1	-1	1	-1	-1	NA	-1	0	-1	NA	-1	-1	1	-1	-1	0	-1
g9	NA	-1	-1	1	NA	NA	NA	1	0	1	1	-1	NA	-1	NA	NA	0	0	0	0
g10	1	NA	NA	NA	1	-1	1	0	NA	0	NA	NA	1	1	0	0	0	-1	1	NA
g11	0	0	1	1	-1	1	-1	0	0	NA	1	1	NA	NA	1	0	NA	-1	0	0
g12	NA	1	1	NA	0	0	NA	NA	-1	1	1	1	0	0	NA	-1	0	0	-1	NA
g13	NA	NA	0	-1	NA	NA	NA	-1	1	NA	NA	-1	NA	1	NA	-NA	-1	NA	NA	0
g14	-1	-1	-1	NA	0	1	-1	0	1	0	1	-1	NA	1	1	-1	0	NA	-1	NA
g15	0	-1	0	NA	1	0	-1	1	NA	NA	1	NA	1	0	0	1	NA	0	-1	0

Version NA70

	m1	m2	m3	m4	m5	m6	m7	m8	m9	m10	m11	m12	m13	m14	m15	m16	m17	m18	m19	m20
g1	NA	NA	NA	-1	NA	NA	-1	1	1	NA	NA	-1	NA	0	NA	NA	-1	-1	NA	0
g2	NA	0	NA	NA	1	NA	1	NA	NA	NA	NA	NA	NA	1	NA	NA	1	-1	1	NA
g3	1	NA	1	NA	NA	0	-1	NA	1	NA	NA	NA	NA	1	0	NA	1	NA	-1	NA
g4	NA	NA	NA	-1	0	NA	NA	0	-1	1	NA	-1	0	-1	NA	0	NA	NA	NA	NA
g5	1	1	NA	NA	-1	-1	0	1	NA	NA	-1	-1	0	0	1	-1	-1	0	NA	NA
g6	1	NA	0	NA	-1	NA	1	NA	0	NA	NA	1	0	-1	NA	NA	-1	1	NA	-1
g7	NA	NA	NA	-1	1	1	-1	NA	NA	0	NA	NA	1	1	NA	1	-1	NA	NA	0
g8	1	0	0	-1	NA	NA	NA	NA	-1	NA	0	-1	NA	NA	-1	1	NA	-1	NA	NA
g9	NA	NA	-1	NA	-1	NA	0	1	0	1	NA	NA	1	-1	NA	NA	0	NA	NA	NA
g10	1	1	NA	NA	NA	-1	NA	NA	NA	0	1	NA	NA	1	NA	NA	0	-1	NA	NA
g11	0	NA	NA	1	-1	NA	-1	0	0	NA	NA	NA	NA	NA	NA	NA	0	-1	0	NA
g12	-1	NA	1	-1	NA	NA	0	NA	-1	1	NA	NA	0	0	1	NA	0	NA	-1	1
g13	NA	-1	0	NA	0	0	1	NA	NA	NA	0	-1	0	1	NA	NA	-1	0	NA	NA
g14	NA	NA	NA	NA	0	NA	NA	NA	1	NA	NA	NA	NA	1	1	-1	0	1	-1	NA
g15	0	NA	NA	-1	1	NA	-1	1	NA	NA	1	NA	1	0	0	NA	NA	0	-1	0

Figure S1 Illustration of example dataset versions NA20, NA50, and NA70
Simulated missing values are depicted in black. Rows (g1-g15) are individual genotypes and columns (m1-m20) are markers. Versions NA20, NA50, and NA70, have up to 20%, 50% and 70% missing data per marker respectively.

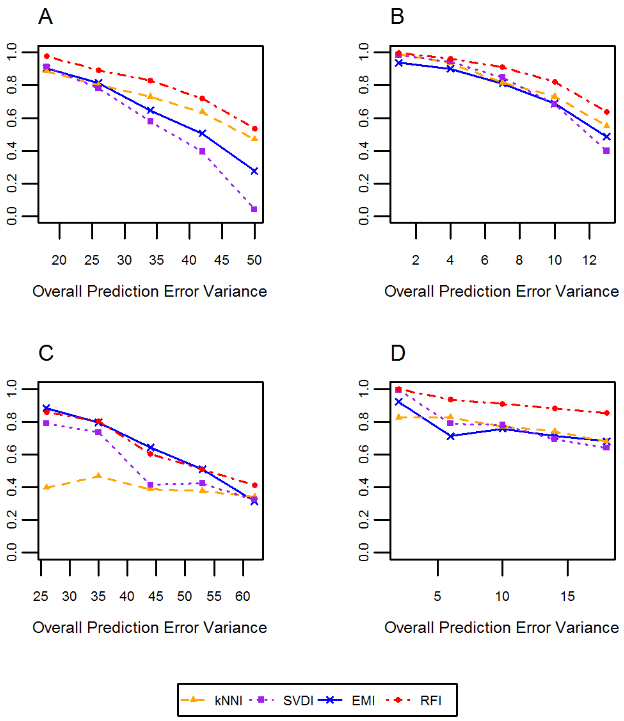

Figure S2 Relationship between the overall expected prediction error variance (PEV) and $\overline{\overline{R_i^2}}$
The median $\overline{\overline{R_i^2}}$ obtained for a given PEV value is plotted for each dataset: (A) Cornell winter wheat (WW), (B) CIMMYT elite spring wheat (SW), (C) CIMMYT drought tolerant maize (DTM), (D) North American barley (NAB). Each color and symbol represents a different imputation method: k-nearest neighbors imputation (kNNI, orange triangles), singular value decomposition imputation (SVDI, purple squares), random forest regression imputation (RFI, red circles), and expectation maximization imputation (EMI, blue crosses).

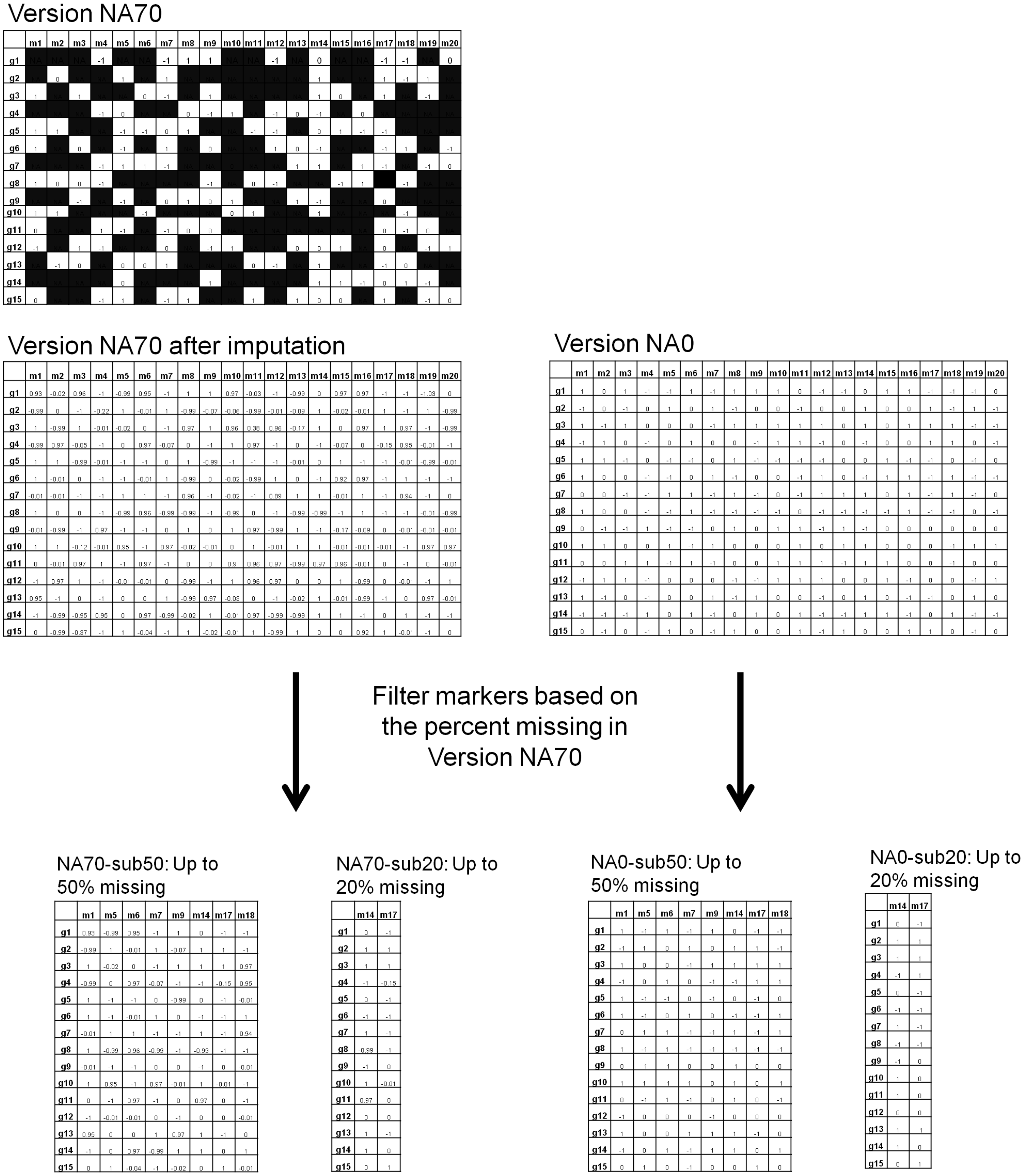

Figure S3 Illustration of the construction of marker sets used to determine the effect of excluding sparse marker data on the genomic selection accuracy

Simulated missing values are depicted in black. Rows (g1-g15) are individual genotypes and columns (m1-m20) are markers. For each population the marker set version NA70 with up to 70% simulated missing data per marker was used and for each marker the percent missing was calculated. This marker set was then imputed with mean imputation, k-nearest neighbors imputation, singular value decomposition imputation, random forest imputation, and expectation maximization imputation. Markers in the imputed sets were then filtered based on their percent missing in version NA70 to create a subset of markers which had up to 20% missing: NA70-sub20, and up to 50% missing: NA70-sub50. For comparison, markers in the original marker set, NA0 were filtered based on their percent missing in version NA70.

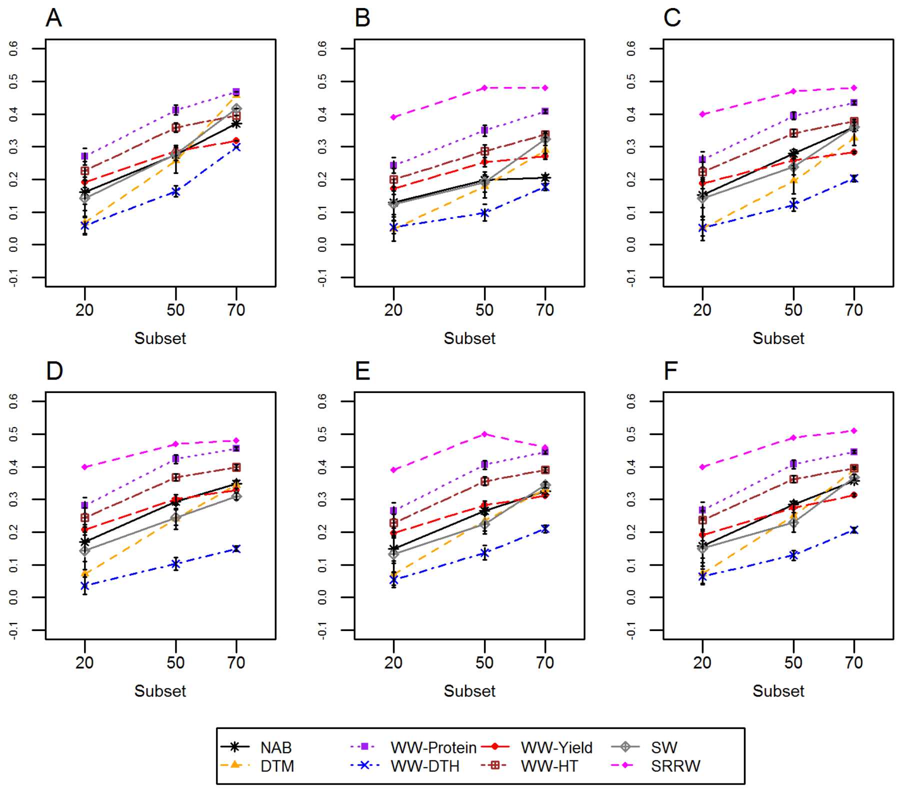

Figure S4 The effect of excluding sparse marker data on the genomic selection accuracy
Mean prediction accuracies obtained with different subsets of the NA70 dataset versions, which had up to 70% missing data per marker, are shown. The subsets compared were NA70-sub20, NA70-sub50, and NA70-sub70 (B-F), these marker sets contained markers with up to 20%, 50% and 70% missing values respectively. Prediction accuracies were also obtained with subsets of the NA0 data set version, which had up to 0% missing data per marker. These subsets were: NA0-sub20, NA0-sub50, and NA0-sub70 (A) and they consisted of the same set of markers as versions NA70-sub20, NA70-sub50, and NA70-sub70 respectively. The imputation methods used were (B) mean imputation (MNI), (C) k nearest neighbors imputation (kNNI), (D) singular value decomposition imputation (SVDI), (E) expectation maximization imputation (EMI), and (F) random forest imputation (RFI). In each panel prediction accuracies are shown for the population-traits: North American barley (NAB; black stars), CIMMYT drought tolerant maize (DTM; orange triangles), Cornell winter wheat (WW)-protein (purple squares), Cornell winter wheat-days to heading (WW-DTH; blue crosses), Cornell winter wheat (WW)-yield (red circles), Cornell winter wheat-height (WW-HT; brown squares), CIMMYT elite spring wheat (SW; grey open squares), stem rust resistant wheat (SRRW; pink diamonds). Error bars depict standard errors.

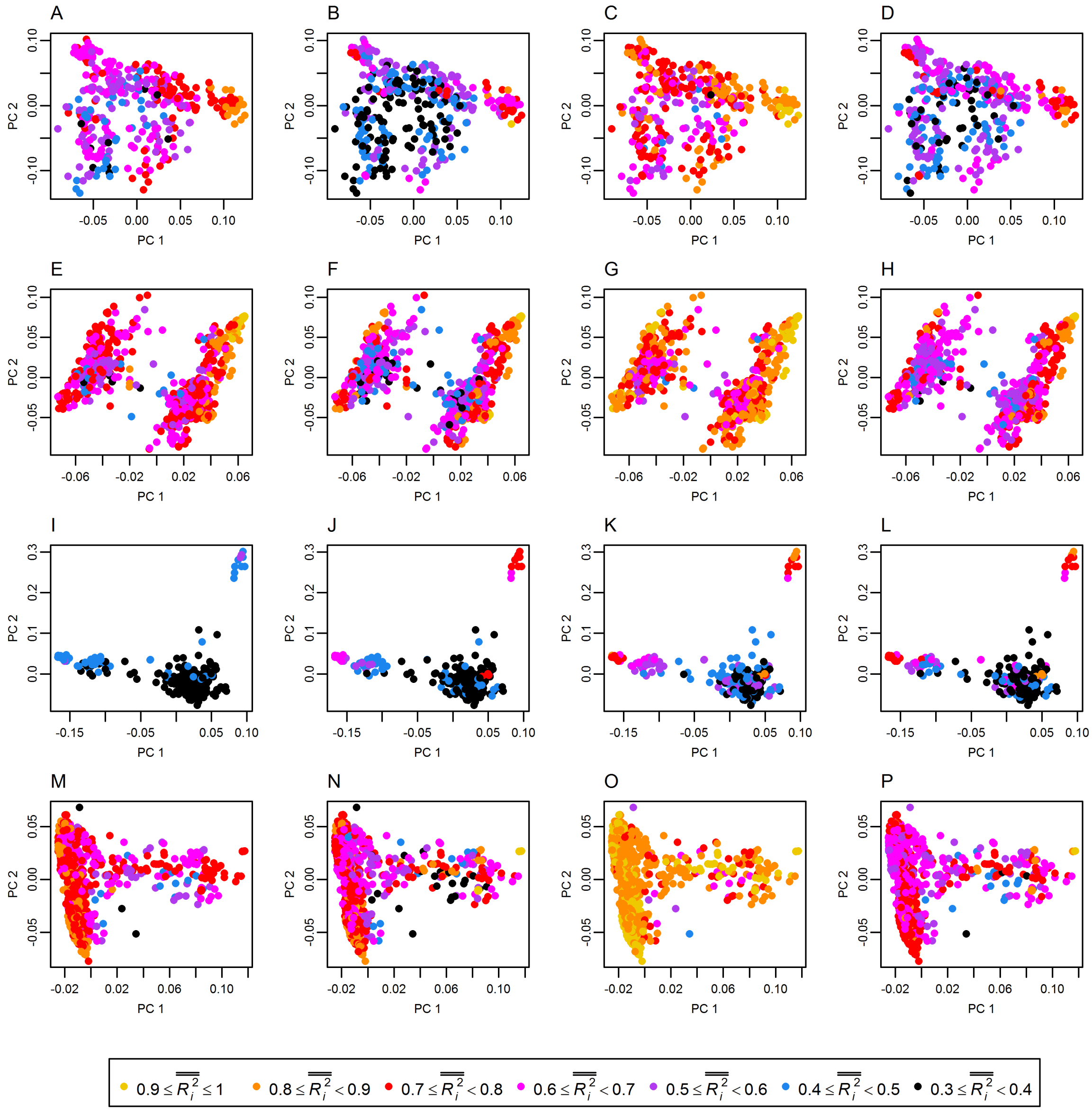

Figure S5 Heterogeneity of accuracies across population sub-groups
In each panel, principal component (PC) 2 *vs.* PC 1 of genotypic data is plotted to show population sub-groups. Each panel corresponds to a dataset-imputation method combination. Individuals are color coded according to their overall average imputation accuracy on an individual genotype basis, $\overline{\overline{R_i^2}}$. The colors yellow, orange, red, pink, purple, blue, and black correspond to $0.9 \le \overline{\overline{R_i^2}} \le 1$, $0.8 \le \overline{\overline{R_i^2}} < 0.9$, $0.7 \le \overline{\overline{R_i^2}} < 0.8$, $0.6 \le \overline{\overline{R_i^2}} < 0.7$, $0.5 \le \overline{\overline{R_i^2}} < 0.6$, $0.4 \le \overline{\overline{R_i^2}} < 0.5$, $0.3 \le \overline{\overline{R_i^2}} < 0.4$ respectively. Panels A-D correspond to the Cornell winter wheat (WW) data imputed with k nearest neighbors imputation (kNNI; A), singular value decomposition imputation (SVDI; B), random forest imputation (RFI, C), expectation maximization imputation (EMI, D). Panels E-H correspond to the CIMMYT elite spring wheat data imputed with kNNI (E), SVDI (F), RFI (G), EMI (H). Panels I-L correspond to the CIMMYT drought tolerant maize data imputed with kNNI (I), SVDI (J), RFI (K), EMI (L). Panels M-P correspond to the North American barley data imputed with kNNI (M), SVDI (N), RFI (O), EMI (P).

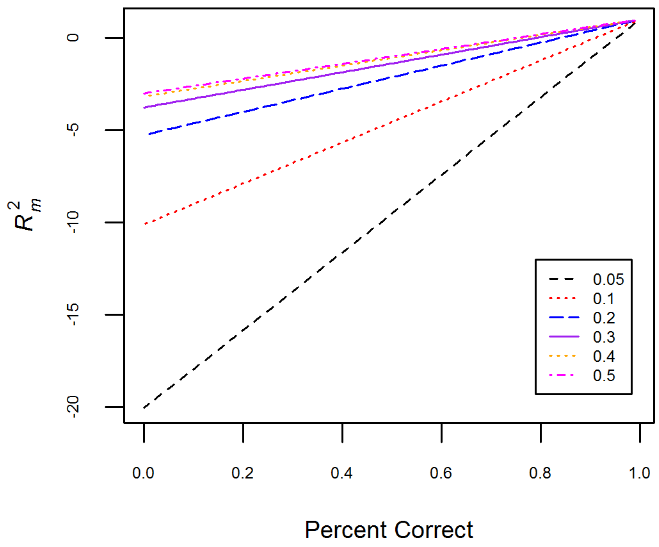

Figure S6 The relationship between imputation accuracy measured as R_m^2 and measured as percent correct for different minor allele frequencies

For each minor allele frequency value: 0.05, 0.1, 0.2, 0.3, 0.4 and 0.5, which is depicted in black, red, blue purple ,orange and magenta respectively, the relationship between the R_m^2 and the percent correct is shown.

File S1

Supporting Text

METHODS

Optimal k value estimation for kNNI and SVDI

Optimal k values for kNNI and SVDI were estimated for the first replicate of each of the 15 datasets and these estimates were used for all remaining replicates. Optimal k values were estimated using 10-fold cross validation. For this procedure a set of k values: 1, 5, 10, 15, 20, 25 were chosen for initial evaluation. For each proposed value of k, a 10-fold cross validation was used to compute the accuracy in terms of median R_m^2. If the largest k value in the initial set of k values was found to be optimal, a new set of larger k values was evaluated. The values in the interval between two k values leading to the highest cross validation accuracy were then selected for the second round of k value evaluation. In this second round, the k value leading to the highest accuracy was considered to be optimal. This process was repeated until a k value leading to maximum cross validation accuracy was determined. To compute the cross validated accuracy, 1) 10 independent sets of non-missing data-points were identified, 2) set one data-points were masked, 3) either kNNI or SVDI was completed using the k value to be evaluated, and 4) steps 2 and 3 were repeated for all 10 sets. The median R_m^2 between the initial dataset and the dataset post-imputation of all 10 sets was used as the evaluation of cross validation accuracy.

Equivalent percent correct calculation

For each marker, 1001 marker genotype vectors, with the marker's MAF were simulated. Each vector had a length of 1000. One of the vectors was selected as the true genotype, and the remaining 1000 were simulated to have different percent correct values, ranging from 0.01 to 100, with an interval of 0.1 between consecutive percent correct values. For each of the 1000 vectors with known percent correct values, R_m^2 was calculated. Then the vector with the R_m^2 value closest to the R_m^2 value for the marker of interest was identified, and that vector's known percent correct value was used as the equivalent percent correct value for the marker of interest.

Genomic selection accuracy calculations (continued)

For the WW, SW, DTM and SRRW datasets the breeding values used for GS model training and validation consisted of best linear unbiased predictors (BLUPs) of the phenotypic values for the genotyped individuals. For the NAB dataset, an individual's phenotypic value *per se* was used as its breeding value. The GS accuracies for all marker set-imputation method

combinations were calculated for a single trait with the SW, DTM, NAB, and SRRW datasets and for four traits with the WW dataset. (The traits that were used are listed in the section describing the original datasets). For more details on the BLUP calculations refer to Heffner et al. (2011) for the WW data and to Crossa et al. (2010) for the SW and DTM data. To compute BLUPs of the phenotypes for the SRRW data the mixed model:

$$Y = X\beta + Z\mu + \varepsilon$$

was fit to the data. Y was the vector of phenotypic observations, β was the vector of site effects treated as fixed effects, μ was the vector of genotype effects treated as random effects, X and Z were the design matrices relating the observations in Y to β and μ.

A 10-fold cross validation was used to compute GS accuracy. This consisted of 1) splitting the dataset into 10 sets, 2) training the model with 9 sets and predicting the remaining set, and 3) repeating steps one and two until predicted values have been calculated for all the individuals. The accuracy was defined as the Pearson's correlation between the breeding values estimated with phenotype and the genomic estimated breeding values (GEBVs). For all versions; NA0, NA20, NA50, and NA70, of a given dataset, individuals were assigned to specific sets that were held constant across all replicates, missing data levels, and traits in order to remove variation in predicted values that would arise due to sampling. This enabled direct comparison of the impact of the different imputation methods and missing data levels on the GS accuracy.

Ridge-Regression (RR, Whittaker et al., 2000) and Bayesian LASSO (BL, de los Campos et al., 2009) were the two prediction models used for computing GS accuracies. For both RR and BL, marker effects were first estimated using the training set. These marker effect estimates and the genotypes of the validation individuals were used to calculate the GEBVs which were defined as the sum of each individual's marker effects. RR assumes that all marker effects are sampled from the same normal distribution with zero mean and variance that is estimated by maximum likelihood. With BL, the variance of the marker effect sampling distribution is unique for each marker. This leads to more and less shrinkage on small- and large-effect markers, respectively. We implemented RR in R (R Development Core Team, 2011). The package 'emma' (Kang et al., 2008) was used to estimate the variance components by maximum likelihood. BL was implemented in the R package BLR (de los Campos and Perez Rodriguez, 2010). The parameter values were set to those suggested by Perez et al. 2010 (Pérez et al., 2010). Marker effect estimations were based on 40,000 iterations of sampling after a burn in period of 20,000 iterations. Trace plots of the variance parameters were inspected to check for convergence.

Effect of excluding sparse marker data on the genomic selection accuracy

In order to determine if markers with a large proportion of missing data should be included rather than filtered from the dataset, we assessed the effect of excluding sparse markers, those with a large proportion of missing data points, on the GS

accuracy. Subsets of the NA70 versions of each dataset were used to calculate GS accuracies after imputation with each method. One of the two subsets contained markers that had up to 20% missing data per-marker before imputation, referred to as NA70-sub20. The second subset contained markers that had up to 50% missing data before imputation, referred to as NA70-sub50. The marker set containing markers with up to 70% missing data (which includes all the markers) is referred to as NA70-sub70. For comparison, the original datasets with no simulated missing data were also subsetted so they would contain the same markers as the NA70-sub20 and NA70-sub50, and NA70-sub70 datasets, these marker sets are referred to as NA0-sub20 and NA0-sub50, NA0-sub70. The numbers of markers in each of the marker sets are listed in Table S1, and an example of the marker sets is illustrated in Figure S2. GS accuracy for each marker set-imputation method combination was calculated using RR. For each imputation method, the differences in GS accuracy between versions NA70-sub20, NA70-sub50, and NA70-sub70 were examined to determine how the GS accuracy is affected by including markers with over 20% missing values and with over 50% missing values after applying each imputation method. GS accuracy was also obtained using versions NA0-sub20, NA0-sub50, and NA0-sub70 to determine how the marker subsets affect the GS accuracy when the true genotypic data is known.

Fst calculation

Using each original dataset, individuals were classified into clusters using model based hierarchical agglomerative clustering described by Fraely and Raftery (2002) implemented using the R package 'mclust' (Fraley et al. 2012). The multivariate normal mixture models evaluated were spherical equal volume (EII), spherical unequal volume (VII), diagonal equal volume and shape (EEI), diagonal equal volume, varying shape (EVI), diagonal varying volume, equal shape (VEI), and diagonal varying volume and shape (VVI). The number of clusters evaluated for each model was 1-15. For each dataset the optimal model and number of clusters was chosen according to the Bayesian information criterion (BIC). For all datasets the optimal model was VEI and the optimal number of clusters was 5, 3, 4, and 5 for the WW, SW, DTM, and NAB datasets respectively.

After individuals were classified into clusters, an Fst value for each marker was calculated to determine its level of differentiation due to genetic structure, or in other words, the amount its variance explained by population structure. Fst was calculated as:

$$\mathrm{Fst} = \frac{\overline{p^2} - \bar{P}^2}{\bar{P}(1 - \bar{P})}$$

where $\overline{p^2}$ is the weighted average of the squared allele frequency across subpopulations for one (arbitrary) allele, and $\bar{P}$ is the weighted average allele frequency across the subpopulations for that same allele (Weir and Cockerham, 1984).

RESULTS

Optimal k values for k nearest neighbors and singular value decomposition imputation

The optimal k values for KNNI and SVDI for each dataset and dataset version are listed in Table S1. The optimal k values for KNNI were low, usually between 2 and 4 for most datasets and dataset versions. Compared to the other datasets, optimal k values for the DTM dataset were more than 10 times larger. The optimal KNNI k value for the SRRW dataset version NA70 was also disproportionally higher than the other dataset and dataset versions. The optimal k vales for SVDI varied depending on the dataset and always decreased as the level of missing data increased. It appeared that higher levels of LD between marker pairs and greater numbers of markers favored larger optimal k-values for SVDI.

Effect of excluding sparse marker data on the genomic selection accuracy

For all imputation methods, there was generally a sharp increase in GS accuracy across the NA70-sub20, and NA70-sub50 versions, and a slight increase across the NA70-sub50 and NA70-sub70 versions (Figure S3, panels B-F) indicating that excluding sparse marker data almost always lead to decreased accuracy especially when a more stringent percent missing threshold was used to filter markers. We found that filtering out the same marker sets when the true data was known had an even larger effect on the GS accuracy (Figure S3 panel A), indicating that marker density was a factor limiting the GS accuracy in these populations. Had marker density not been limiting, including sparse markers may not have lead to increased accuracy.

REFERENCES

de los Campos, G., H. Naya, D. Gianola, J. Crossa, A. Legarra *et al.*, 2009 Predicting quantitative traits with regression models for dense molecular markers and pedigree. Genetics 182: 375-85.

de los Campos, G., and P. Perez Rodriguez, 2010 BLR: Bayesian linear regression. R package versión 1.2. Avaliable at: http://CRAN.R-project.org/package=BLR/ (verified 30 July 2012).

Crossa, J., G. de los Campos, P. Pérez, D. Gianola, J. Burgueño *et al.*, 2010 Prediction of genetic values of quantitative traits in plant breeding using pedigree and molecular markers. Genetics 186: 713-724.

Fraley, C., and A.E. Raftery, 2002 Model-based clustering, discriminant analysis, and density estimation. J. Amer. Statist. Assoc. 97: 611-631.

Fraley, C., A.E. Raftery, T.B. Murphey, and L. Scrucca, 2012 mclust Version 4 for R: normal mixture modeling for model-based clustering, classification, and density estimation technical report No. 597, Department of Statistics, University of Washington

Heffner, E. L., J.-L. Jannink, and M. E. Sorrells, 2011 Genomic selection accuracy using multifamily prediction models in a wheat breeding program. Plant Gen. 4: 65-75.

Kang, H. M., N. A. Zaitlen, C. M. Wade, A. Kirby, D. Heckerman *et al.*, 2008 Efficient control of population structure in model organism association mapping. Genetics 178: 1709-1723.

Pérez, P., G. D. L. Campos, J. Crossa, D. Gianola, and G. de los Campos, 2010 Genomic-enabled prediction based on molecular markers and pedigree using the Bayesian linear regression package in R. Plant Genome 3: 106-116.

R Development Core Team, 2011 R: A Language and Environment for Statistical Computing, Vienna. Available at: http://www.r-project.org/ (verified 30 July 2012).

Weir, B. S., and C. C. Cockerham, 1984 Estimating F-statistics for the analysis of population structure. Evolution 6: 1358-1370.

Whittaker, J. C., R. Thompson, and M. C. Denham, 2000 Marker-assisted selection using ridge regression. Genet. Res. 75: 249-252.

Files S2-S6

File S2: Cornell winter wheat dataset

File S3: Stem rust resistant wheat dataset version NA20

File S4: Stem rust resistant wheat dataset version NA50

File S5: Stem rust resistant wheat dataset version NA70

File S6: R code and workspace for example imputation

Files S2-S6 are available for download at http://www.genetics.org/lookup/suppl/doi:10.1534/g3.112.005363/-/DC1.

Table S1 Optimal k values for KNNI[a] and SVDI[b] used across all replicates

Dataset[c]	Version[d]	KNNI	SVDI
WW	NA20	3	78
	NA50	3	51
	NA70	4	19
SW	NA20	2	88
	NA50	3	89
	NA70	4	33
DTM	NA20	51	10
	NA50	39	9
	NA70	49	5
NAB	NA20	4	190
	NA50	4	117
	NA70	4	50
SRRW	NA20	3	81
	NA50	4	36
	NA70	66	1

[a] KNNI: k-nearest neighbors imputation
[b] SVDI: singular value decomposition imputation
[c] WW: Cornell winter wheat, SW: CIMMYT elite spring wheat, DTM: CIMMYT drought tolerant maize, NAB: North American barley, SRRW: CIMMYT stem rust resistant wheat
[d]NA20: up to 20% missing data per marker, NA50: up to 50% missing data per marker, NA70: up to 70% missing data per marker

Table S2 Description of datasets used to test the effect of excluding sparse marker data on the genomic selection accuracy

Dataset[a]	Version[b]	Mean number of markers
WW	NA70-sub20	20
	NA70-sub50	119
	NA70-sub70	1158
SW	NA70-sub20	23
	NA70-sub50	112
	NA70-sub70	1279
DTM	NA70-sub20	18
	NA70-sub50	99
	NA70-sub70	1135
NAB	NA70-sub20	37
	NA70-sub50	185
	NA70-sub70	2146
SRRW	NA70-sub20	34
	NA70-sub50	169
	NA70-sub70	2014

[a] WW: Cornell winter wheat, SW: CIMMYT elite spring wheat, DTM: CIMMYT drought tolerant maize, NAB: North American barley, SRRW: CIMMYT stem rust resistant wheat
[b] Version NA70-sub20: up to 70% missing data per marker was simulated, and markers were discarded if they had over 20% missing data. Version NA70-sub50: up to 70% missing data per marker was simulated, and markers were discarded if they had over 50% missing data. Version NA70-sub70: up to 70% missing data per marker was simulated, and no markers were discarded.
