# Supplementary material for: Imputation of Unordered Markers and the Impact on Genomic Selection Accuracy
Source: G3 (Bethesda). 2013 Mar 1;3(3):427–39. doi: 10.1534/g3.112.005363 (PMC3583451; doi:10.1534/g3.112.005363)
Supplement: Supporting Information [file supp_3.3.427_FigureS1.pdf]

Version NA0

	m1	m2	m3	m4	m5	m6	m7	m8	m9	m10	m11	m12	m13	m14	m15	m16	m17	m18	m19	m20
**g1**	1	0	1	-1	-1	1	-1	1	1	1	0	-1	-1	0	1	1	-1	-1	-1	0
**g2**	-1	0	-1	0	1	0	1	-1	0	0	-1	0	0	1	0	0	1	-1	1	-1
**g3**	1	-1	1	0	0	0	-1	1	1	1	1	1	0	1	0	1	1	1	-1	-1
**g4**	-1	1	0	-1	0	1	0	0	-1	1	1	-1	0	-1	0	0	1	1	0	-1
**g5**	1	1	-1	0	-1	-1	0	1	-1	-1	-1	-1	0	0	1	-1	-1	0	-1	0
**g6**	1	0	0	-1	-1	0	1	-1	0	0	-1	1	0	-1	1	1	-1	1	-1	-1
**g7**	0	0	-1	-1	1	1	-1	1	-1	0	-1	1	1	1	0	1	-1	1	-1	0
**g8**	1	0	0	-1	-1	1	-1	-1	-1	-1	0	-1	-1	-1	-1	1	-1	-1	0	-1
**g9**	0	-1	-1	1	-1	-1	0	1	0	1	1	-1	1	-1	0	0	0	0	0	0
**g10**	1	1	0	0	1	-1	1	0	0	0	1	0	1	1	0	0	0	-1	1	1
**g11**	0	0	1	1	-1	1	-1	0	0	1	1	1	-1	1	1	0	0	-1	0	0
**g12**	-1	1	1	-1	0	0	0	-1	-1	1	1	1	0	0	1	-1	0	0	-1	1
**g13**	1	-1	0	-1	0	0	1	-1	1	0	0	-1	0	1	0	-1	-1	0	1	0
**g14**	-1	-1	-1	1	0	1	-1	0	1	0	1	-1	-1	1	1	-1	0	1	-1	-1
**g15**	0	-1	0	-1	1	0	-1	1	0	0	1	-1	1	0	0	1	1	0	-1	0

Version NA20

	m1	m2	m3	m4	m5	m6	m7	m8	m9	m10	m11	m12	m13	m14	m15	m16	m17	m18	m19	m20
**g1**	1	0	1	-1	-1	1	-1	1	1	1	0	-1	-1	0	1	1	-1	-1	-1	0
**g2**	-1	NA	-1	0	1	0	1	-1	0	0	-1	0	0	NA	-1	0	1	NA	-1	-1
**g3**	1	-1	NA	-1	NA	-1	-1	1	1	1	1	1	0	1	0	NA	-1	1	-1	-1
**g4**	NA	1	0	-1	0	NA	-1	0	-1	NA	-1	-1	0	-1	0	0	1	1	0	-1
**g5**	1	1	-1	0	-1	-1	0	NA	-1	-1	-1	-1	0	0	1	-1	NA	-1	-1	0
**g6**	1	0	0	-1	NA	-1	NA	-1	0	0	-1	1	NA	-1	1	1	-1	1	-1	-1
**g7**	0	0	-1	-1	1	1	-1	1	-1	0	-1	NA	-1	1	0	1	-1	1	-1	0
**g8**	1	0	NA	-1	-1	1	-1	-1	-1	NA	-1	-1	-1	-1	NA	NA	-1	-1	0	NA
**g9**	NA	-1	-1	1	-1	NA	-1	1	0	1	1	-1	1	-1	0	0	0	NA	-1	0
**g10**	1	1	0	0	1	-1	NA	-1	NA	-1	1	0	1	1	0	0	0	-1	1	1
**g11**	0	0	1	1	-1	NA	-1	0	0	1	1	1	-1	1	1	0	NA	-1	0	0
**g12**	-1	1	1	-1	0	0	NA	-1	-1	1	NA	-1	0	NA	-1	-1	0	0	-1	NA
**g13**	1	-1	0	-1	0	0	1	-1	1	NA	-1	-1	0	1	0	-1	NA	-1	1	0
**g14**	-1	-1	NA	-1	0	1	-1	0	1	0	1	-1	NA	-1	1	-1	0	1	-1	-1
**g15**	0	-1	0	-1	1	0	-1	1	0	0	NA	-1	1	0	0	NA	-1	0	-1	0

Version NA50

	m1	m2	m3	m4	m5	m6	m7	m8	m9	m10	m11	m12	m13	m14	m15	m16	m17	m18	m19	m20
**g1**	NA	0	NA	NA	-1	1	-1	NA	1	1	NA	NA	-1	0	NA	1	NA	-1	NA	NA
**g2**	NA	0	-1	0	NA	0	NA	-1	NA	0	-1	0	0	NA	0	NA	1	-1	NA	NA
**g3**	1	-1	1	0	0	NA	-1	1	1	NA	1	NA	NA	1	0	1	NA	NA	-1	NA
**g4**	-1	1	NA	NA	0	1	0	0	-1	1	1	-1	0	-1	0	0	1	1	0	-1
**g5**	NA	NA	-1	0	NA	-1	NA	1	NA	-1	-1	-1	NA	NA	1	NA	-1	0	NA	0
**g6**	1	0	0	-1	-1	0	1	NA	NA	NA	-1	1	0	-1	1	1	NA	NA	NA	-1
**g7**	NA	NA	NA	NA	1	1	-1	1	NA	0	-1	1	1	NA	0	1	NA	1	-1	NA
**g8**	1	0	0	-1	-1	1	-1	-1	NA	-1	0	-1	NA	-1	-1	1	-1	-1	0	-1
**g9**	NA	-1	-1	1	NA	NA	NA	1	0	1	1	-1	NA	-1	NA	NA	0	0	0	0
**g10**	1	NA	NA	NA	1	-1	1	0	NA	0	NA	NA	1	1	0	0	0	-1	1	NA
**g11**	0	0	1	1	-1	1	-1	0	0	NA	1	1	NA	NA	1	0	NA	-1	0	0
**g12**	NA	1	1	NA	0	0	NA	NA	-1	1	1	1	0	0	NA	-1	0	0	-1	NA
**g13**	NA	NA	0	-1	NA	NA	NA	-1	1	NA	NA	-1	NA	1	NA	NA	-1	NA	NA	0
**g14**	-1	-1	-1	NA	0	1	-1	0	1	0	1	-1	NA	1	1	-1	0	NA	-1	NA
**g15**	0	-1	0	NA	1	0	-1	1	NA	NA	1	NA	1	0	0	1	NA	0	-1	0

Version NA70

	m1	m2	m3	m4	m5	m6	m7	m8	m9	m10	m11	m12	m13	m14	m15	m16	m17	m18	m19	m20
**g1**	NA	NA	NA	-1	NA	NA	-1	1	1	NA	NA	-1	NA	0	NA	NA	-1	-1	NA	0
**g2**	NA	0	NA	NA	1	NA	1	NA	NA	NA	NA	NA	NA	1	NA	NA	1	-1	1	NA
**g3**	1	NA	1	NA	NA	0	-1	NA	1	NA	NA	NA	NA	1	0	NA	1	NA	-1	NA
**g4**	NA	NA	NA	-1	0	NA	NA	0	-1	1	NA	-1	0	-1	NA	0	NA	NA	NA	NA
**g5**	1	1	NA	NA	-1	-1	0	1	NA	NA	-1	-1	0	0	1	-1	-1	0	NA	NA
**g6**	1	NA	0	NA	-1	NA	1	NA	0	NA	NA	1	0	-1	NA	NA	-1	1	NA	-1
**g7**	NA	NA	NA	-1	1	1	-1	NA	NA	0	NA	NA	1	1	NA	1	-1	NA	NA	0
**g8**	1	0	0	-1	NA	NA	NA	NA	-1	NA	0	-1	NA	NA	-1	1	NA	-1	NA	NA
**g9**	NA	NA	-1	NA	-1	NA	0	1	0	1	NA	NA	1	-1	NA	NA	0	NA	NA	NA
**g10**	1	1	NA	NA	NA	-1	NA	NA	NA	0	1	NA	NA	1	NA	NA	0	-1	NA	NA
**g11**	0	NA	NA	1	-1	NA	-1	0	0	NA	NA	NA	NA	NA	NA	NA	0	-1	0	NA
**g12**	-1	NA	1	-1	NA	NA	0	NA	-1	1	NA	NA	0	0	1	NA	0	NA	-1	1
**g13**	NA	-1	0	NA	0	0	1	NA	NA	NA	0	-1	0	1	NA	NA	-1	0	NA	NA
**g14**	NA	NA	NA	NA	0	NA	NA	NA	1	NA	NA	NA	NA	1	1	-1	0	1	-1	NA
**g15**	0	NA	NA	-1	1	NA	-1	1	NA	NA	1	NA	1	0	0	NA	NA	0	-1	0

Figure S1 Illustration of example dataset versions NA20, NA50, and NA70
Simulated missing values are depicted in black. Rows (g1-g15) are individual genotypes and columns (m1-m20) are markers. Versions NA20, NA50, and NA70, have up to 20%, 50% and 70% missing data per marker respectively.
