# Supplementary material for: Imputation of Unordered Markers and the Impact on Genomic Selection Accuracy
Source: G3 (Bethesda). 2013 Mar 1;3(3):427–39. doi: 10.1534/g3.112.005363 (PMC3583451; doi:10.1534/g3.112.005363)
Supplement: Supporting Information [file supp_3.3.427_FigureS2.pdf]

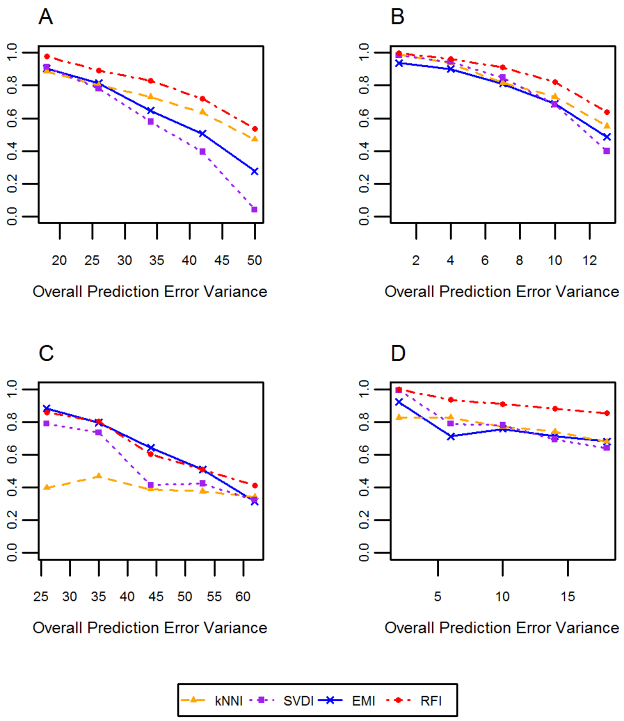

Figure S2 Relationship between the overall expected prediction error variance (PEV) and $\overline{\overline{R_i^2}}$
The median $\overline{\overline{R_i^2}}$ obtained for a given PEV value is plotted for each dataset: (A) Cornell winter wheat (WW), (B) CIMMYT elite spring wheat (SW), (C) CIMMYT drought tolerant maize (DTM), (D) North American barley (NAB). Each color and symbol represents a different imputation method: k-nearest neighbors imputation (kNNI, orange triangles), singular value decomposition imputation (SVDI, purple squares), random forest regression imputation (RFI, red circles), and expectation maximization imputation (EMI, blue crosses).
