# Supplementary material for: Imputation of Unordered Markers and the Impact on Genomic Selection Accuracy
Source: G3 (Bethesda). 2013 Mar 1;3(3):427–39. doi: 10.1534/g3.112.005363 (PMC3583451; doi:10.1534/g3.112.005363)
Supplement: Supporting Information [file supp_3.3.427_FigureS3.pdf]

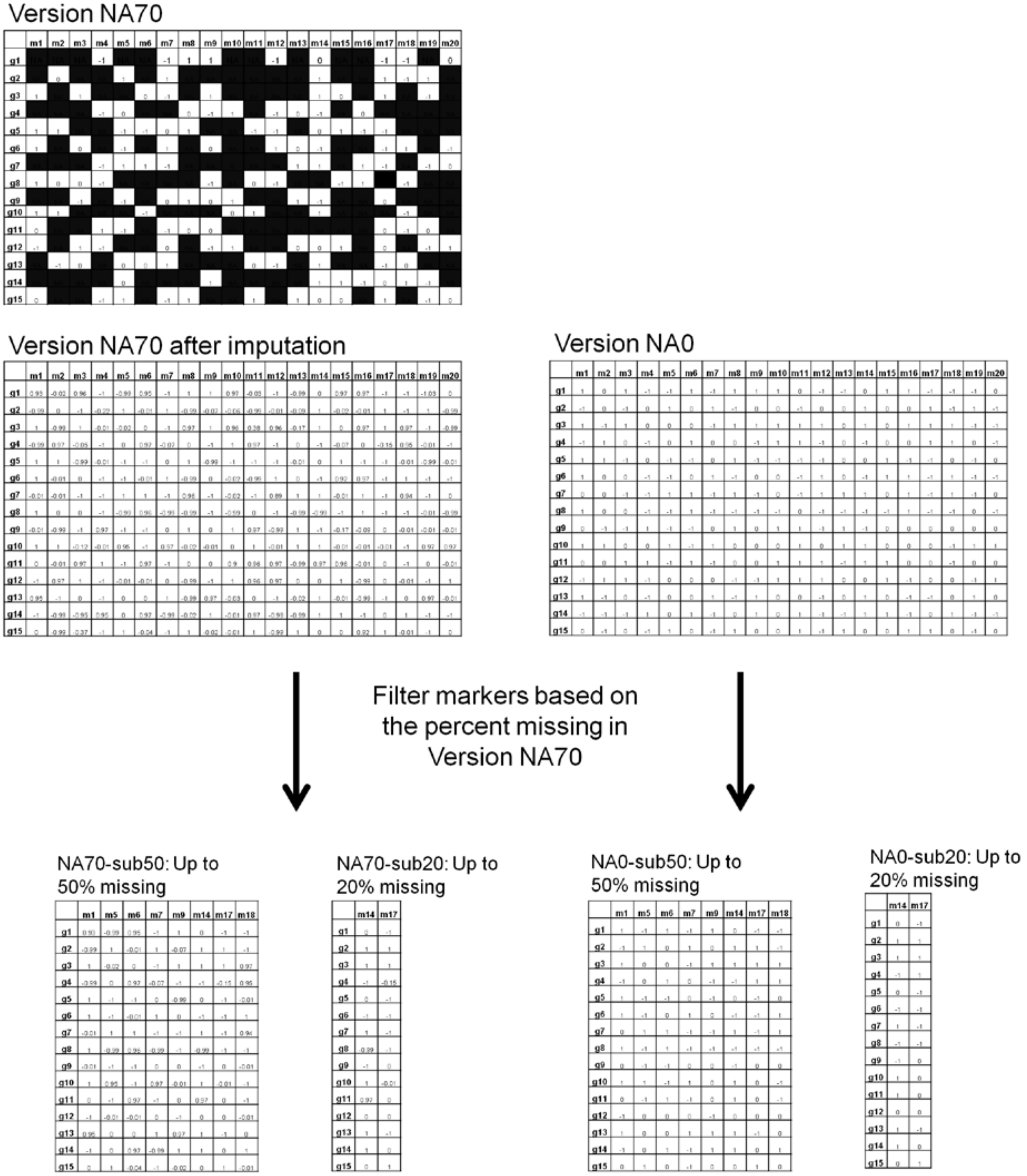

Figure S3 Illustration of the construction of marker sets used to determine the effect of excluding sparse marker data on the genomic selection accuracy

Simulated missing values are depicted in black. Rows (g1-g15) are individual genotypes and columns (m1-m20) are markers. For each population the marker set version NA70 with up to 70% simulated missing data per marker was used and for each marker the percent missing was calculated. This marker set was then imputed with mean imputation, k-nearest neighbors imputation, singular value decomposition imputation, random forest imputation, and expectation maximization imputation. Markers in the imputed sets were then filtered based on their percent missing in version NA70 to create a subset of markers which had up to 20% missing: NA70-sub20, and up to 50% missing: NA70-sub50. For comparison, markers in the original marker set, NA0 were filtered based on their percent missing in version NA70.
