# Supplementary material for: Imputation of Unordered Markers and the Impact on Genomic Selection Accuracy
Source: G3 (Bethesda). 2013 Mar 1;3(3):427–39. doi: 10.1534/g3.112.005363 (PMC3583451; doi:10.1534/g3.112.005363)
Supplement: Supporting Information [file supp_3.3.427_FigureS4.pdf]

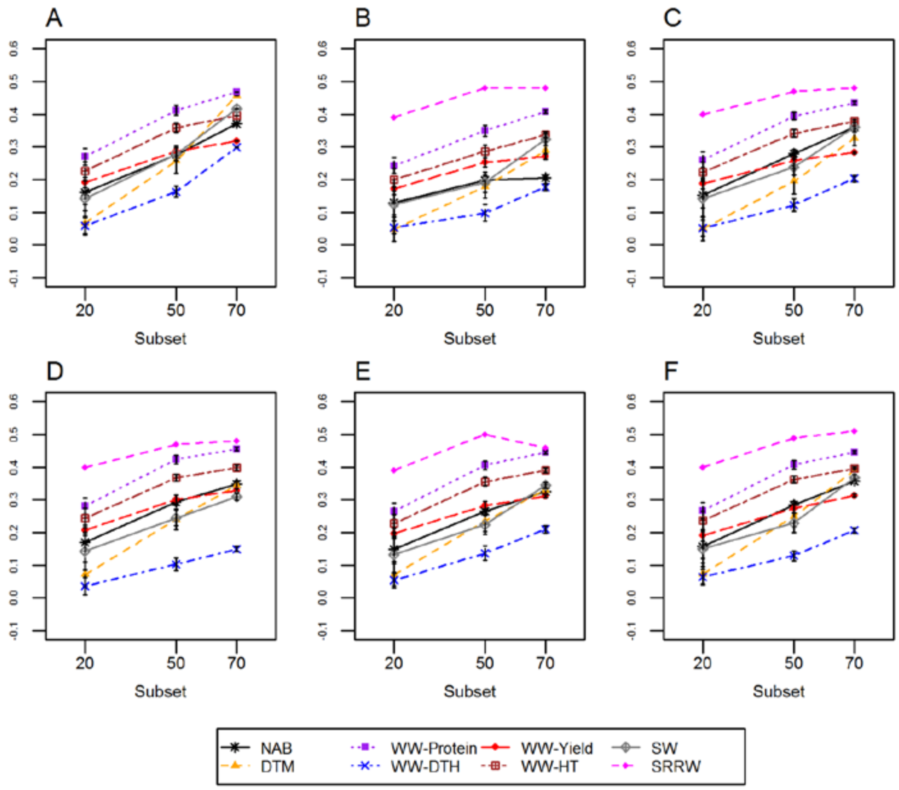

Figure S4 The effect of excluding sparse marker data on the genomic selection accuracy
Mean prediction accuracies obtained with different subsets of the NA70 dataset versions, which had up to 70% missing data per marker, are shown. The subsets compared were NA70-sub20, NA70-sub50, and NA70-sub70 (B-F), these marker sets contained markers with up to 20%, 50% and 70% missing values respectively. Prediction accuracies were also obtained with subsets of the NA0 data set version, which had up to 0% missing data per marker. These subsets were: NA0-sub20, NA0-sub50, and NA0-sub70 (A) and they consisted of the same set of markers as versions NA70-sub20, NA70-sub50, and NA70-sub70 respectively. The imputation methods used were (B) mean imputation (MNI), (C) k nearest neighbors imputation (kNNI), (D) singular value decomposition imputation (SVDI), (E) expectation maximization imputation (EMI), and (F) random forest imputation (RFI). In each panel prediction accuracies are shown for the population-traits: North American barley (NAB; black stars), CIMMYT drought tolerant maize (DTM; orange triangles), Cornell winter wheat (WW)-protein (purple squares), Cornell winter wheat-days to heading (WW-DTH; blue crosses), Cornell winter wheat (WW)-yield (red circles), Cornell winter wheat-height (WW-HT; brown squares), CIMMYT elite spring wheat (SW; grey open squares), stem rust resistant wheat (SRRW; pink diamonds). Error bars depict standard errors.
