# Supplementary material for: Imputation of Unordered Markers and the Impact on Genomic Selection Accuracy
Source: G3 (Bethesda). 2013 Mar 1;3(3):427–39. doi: 10.1534/g3.112.005363 (PMC3583451; doi:10.1534/g3.112.005363)
Supplement: Supporting Information [file supp_3.3.427_FigureS5.pdf]

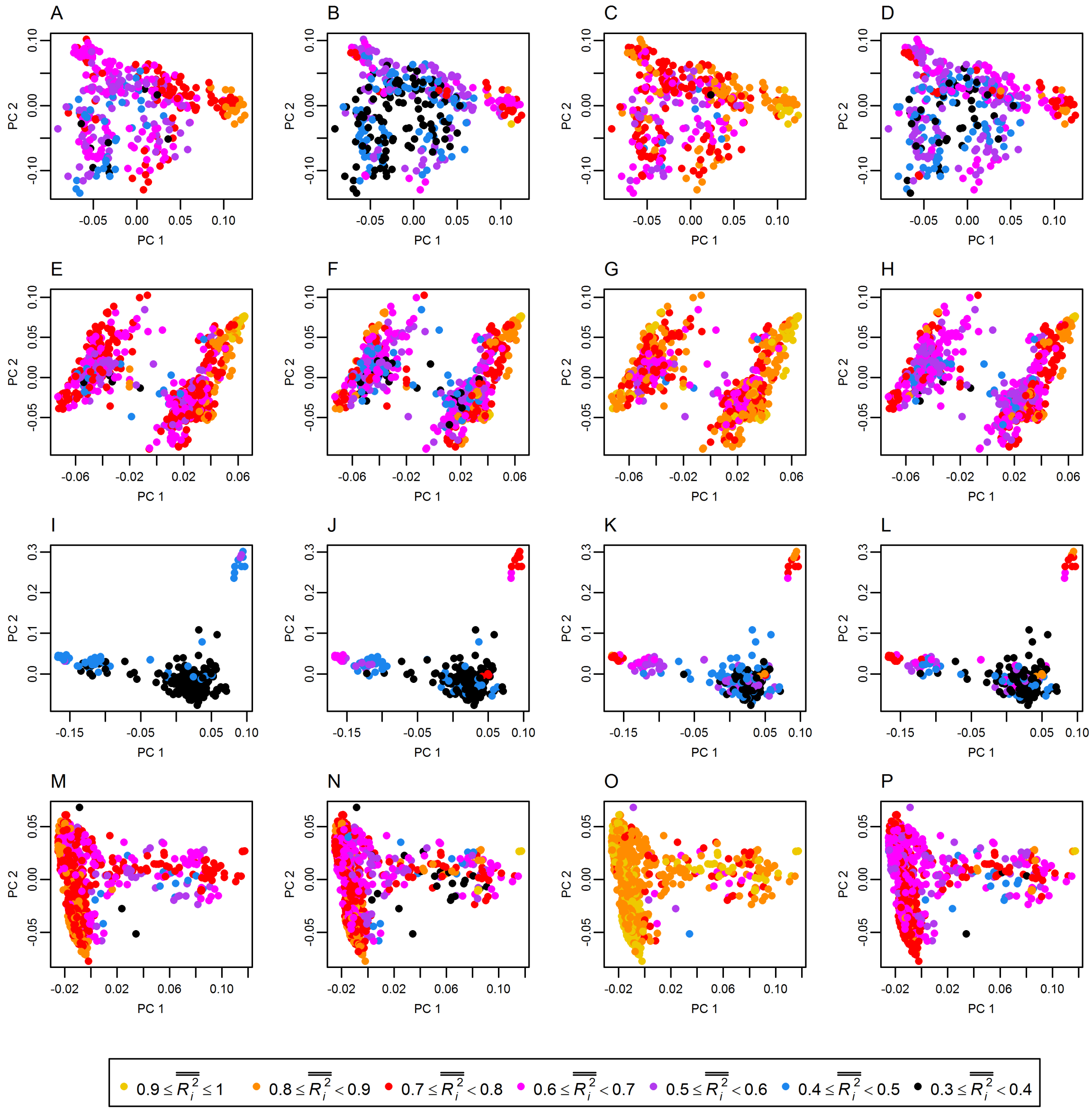


**Figure S5** Heterogeneity of accuracies across population sub-groups
In each panel, principal component (PC) 2 *vs.* PC 1 of genotypic data is plotted to show population sub-groups. Each panel corresponds to a dataset-imputation method combination. Individuals are color coded according to their overall average imputation accuracy on an individual genotype basis, $\overline{\overline{R_i^2}}$. The colors yellow, orange, red, pink, purple, blue, and black correspond to $0.9 \leq \overline{\overline{R_i^2}} \leq 1$, $0.8 \leq \overline{\overline{R_i^2}} < 0.9$, $0.7 \leq \overline{\overline{R_i^2}} < 0.8$, $0.6 \leq \overline{\overline{R_i^2}} < 0.7$, $0.5 \leq \overline{\overline{R_i^2}} < 0.6$, $0.4 \leq \overline{\overline{R_i^2}} < 0.5$, $0.3 \leq \overline{\overline{R_i^2}} < 0.4$ respectively. Panels A-D correspond to the Cornell winter wheat (WW) data imputed with k nearest neighbors imputation (kNNI; A), singular value decomposition imputation (SVDI; B), random forest imputation (RFI, C), expectation maximization imputation (EMI, D). Panels E-H correspond to the CIMMYT elite spring wheat data imputed with kNNI (E), SVDI (F), RFI (G), EMI (H). Panels I-L correspond to the CIMMYT drought tolerant maize data imputed with kNNI (I), SVDI (J), RFI (K), EMI (L). Panels M-P correspond to the North American barley data imputed with kNNI (M), SVDI (N), RFI (O), EMI (P).
