# Supplementary material for: Imputation of Unordered Markers and the Impact on Genomic Selection Accuracy
Source: G3 (Bethesda). 2013 Mar 1;3(3):427–39. doi: 10.1534/g3.112.005363 (PMC3583451; doi:10.1534/g3.112.005363)
Supplement: Supporting Information [file supp_3.3.427_FigureS6.pdf]

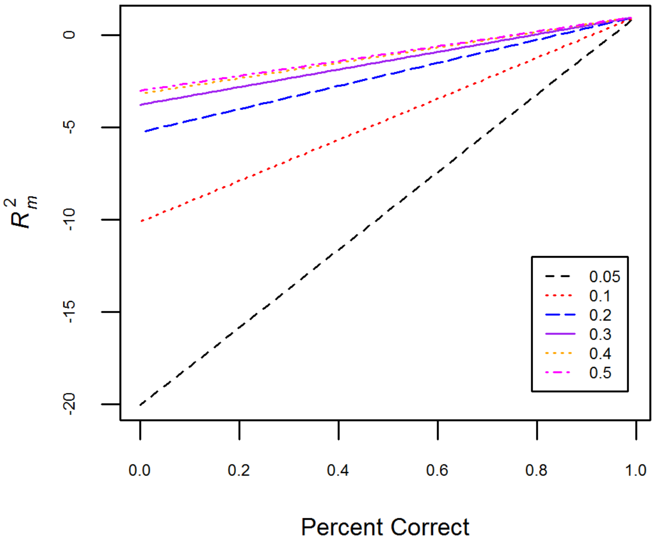

Figure S6 The relationship between imputation accuracy measured as R_m^2 and measured as percent correct for different minor allele frequencies

For each minor allele frequency value: 0.05, 0.1, 0.2, 0.3, 0.4 and 0.5, which is depicted in black, red, blue purple ,orange and magenta respectively, the relationship between the R_m^2 and the percent correct is shown.
