# Supplementary material for: Imputation of Unordered Markers and the Impact on Genomic Selection Accuracy
Source: G3 (Bethesda). 2013 Mar 1;3(3):427–39. doi: 10.1534/g3.112.005363 (PMC3583451; doi:10.1534/g3.112.005363)
Supplement: Supporting Information [file supp_3.3.427_TableS1.pdf]

Table S1 Optimal k values for KNNI[a] and SVDI[b] used across all replicates

Dataset[c]	Version[d]	KNNI	SVDI
WW	NA20	3	78
	NA50	3	51
	NA70	4	19
SW	NA20	2	88
	NA50	3	89
	NA70	4	33
DTM	NA20	51	10
	NA50	39	9
	NA70	49	5
NAB	NA20	4	190
	NA50	4	117
	NA70	4	50
SRRW	NA20	3	81
	NA50	4	36
	NA70	66	1

[a] KNNI: k-nearest neighbors imputation
[b] SVDI: singular value decomposition imputation
[c] WW: Cornell winter wheat, SW: CIMMYT elite spring wheat, DTM: CIMMYT drought tolerant maize, NAB: North American barley, SRRW: CIMMYT stem rust resistant wheat
[d]NA20: up to 20% missing data per marker, NA50: up to 50% missing data per marker, NA70: up to 70% missing data per marker
