# Supplementary material for: Imputation of Unordered Markers and the Impact on Genomic Selection Accuracy
Source: G3 (Bethesda). 2013 Mar 1;3(3):427–39. doi: 10.1534/g3.112.005363 (PMC3583451; doi:10.1534/g3.112.005363)
Supplement: Supporting Information [file supp_3.3.427_TableS2.pdf]

Table S2 Description of datasets used to test the effect of excluding sparse marker data on the genomic selection accuracy

Dataset[a]	Version[b]	Mean number of markers
WW	NA70-sub20	20
	NA70-sub50	119
	NA70-sub70	1158
SW	NA70-sub20	23
	NA70-sub50	112
	NA70-sub70	1279
DTM	NA70-sub20	18
	NA70-sub50	99
	NA70-sub70	1135
NAB	NA70-sub20	37
	NA70-sub50	185
	NA70-sub70	2146
SRRW	NA70-sub20	34
	NA70-sub50	169
	NA70-sub70	2014

[a] WW: Cornell winter wheat, SW: CIMMYT elite spring wheat, DTM: CIMMYT drought tolerant maize, NAB: North American barley, SRRW: CIMMYT stem rust resistant wheat
[b] Version NA70-sub20: up to 70% missing data per marker was simulated, and markers were discarded if they had over 20% missing data. Version NA70-sub50: up to 70% missing data per marker was simulated, and markers were discarded if they had over 50% missing data. Version NA70-sub70: up to 70% missing data per marker was simulated, and no markers were discarded.
