# Supplementary material for: Imputation of Unordered Markers and the Impact on Genomic Selection Accuracy
Source: G3 (Bethesda). 2013 Mar 1;3(3):427–39. doi: 10.1534/g3.112.005363 (PMC3583451; doi:10.1534/g3.112.005363)
Supplement: Supporting Information [file supp_3.3.427_FileS1.pdf]

File S1

Supporting Text

METHODS

Optimal k value estimation for kNNI and SVDI

Optimal k values for kNNI and SVDI were estimated for the first replicate of each of the 15 datasets and these estimates were used for all remaining replicates. Optimal k values were estimated using 10-fold cross validation. For this procedure a set of k values: 1, 5, 10, 15, 20, 25 were chosen for initial evaluation. For each proposed value of k, a 10-fold cross validation was used to compute the accuracy in terms of median R_m^2. If the largest k value in the initial set of k values was found to be optimal, a new set of larger k values was evaluated. The values in the interval between two k values leading to the highest cross validation accuracy were then selected for the second round of k value evaluation. In this second round, the k value leading to the highest accuracy was considered to be optimal. This process was repeated until a k value leading to maximum cross validation accuracy was determined. To compute the cross validated accuracy, 1) 10 independent sets of non-missing data-points were identified, 2) set one data-points were masked, 3) either kNNI or SVDI was completed using the k value to be evaluated, and 4) steps 2 and 3 were repeated for all 10 sets. The median R_m^2 between the initial dataset and the dataset post-imputation of all 10 sets was used as the evaluation of cross validation accuracy.

Equivalent percent correct calculation

For each marker, 1001 marker genotype vectors, with the marker's MAF were simulated. Each vector had a length of 1000. One of the vectors was selected as the true genotype, and the remaining 1000 were simulated to have different percent correct values, ranging from 0.01 to 100, with an interval of 0.1 between consecutive percent correct values. For each of the 1000 vectors with known percent correct values, R_m^2 was calculated. Then the vector with the R_m^2 value closest to the R_m^2 value for the marker of interest was identified, and that vector's known percent correct value was used as the equivalent percent correct value for the marker of interest.

Genomic selection accuracy calculations (continued)

For the WW, SW, DTM and SRRW datasets the breeding values used for GS model training and validation consisted of best linear unbiased predictors (BLUPs) of the phenotypic values for the genotyped individuals. For the NAB dataset, an individual's phenotypic value *per se* was used as its breeding value. The GS accuracies for all marker set-imputation method

combinations were calculated for a single trait with the SW, DTM, NAB, and SRRW datasets and for four traits with the WW dataset. (The traits that were used are listed in the section describing the original datasets). For more details on the BLUP calculations refer to Heffner et al. (2011) for the WW data and to Crossa et al. (2010) for the SW and DTM data. To compute BLUPs of the phenotypes for the SRRW data the mixed model:

$$Y = X\beta + Z\mu + \varepsilon$$

was fit to the data. Y was the vector of phenotypic observations, β was the vector of site effects treated as fixed effects, μ was the vector of genotype effects treated as random effects, X and Z were the design matrices relating the observations in Y to β and μ.

A 10-fold cross validation was used to compute GS accuracy. This consisted of 1) splitting the dataset into 10 sets, 2) training the model with 9 sets and predicting the remaining set, and 3) repeating steps one and two until predicted values have been calculated for all the individuals. The accuracy was defined as the Pearson's correlation between the breeding values estimated with phenotype and the genomic estimated breeding values (GEBVs). For all versions; NA0, NA20, NA50, and NA70, of a given dataset, individuals were assigned to specific sets that were held constant across all replicates, missing data levels, and traits in order to remove variation in predicted values that would arise due to sampling. This enabled direct comparison of the impact of the different imputation methods and missing data levels on the GS accuracy.

Ridge-Regression (RR, Whittaker et al., 2000) and Bayesian LASSO (BL, de los Campos et al., 2009) were the two prediction models used for computing GS accuracies. For both RR and BL, marker effects were first estimated using the training set. These marker effect estimates and the genotypes of the validation individuals were used to calculate the GEBVs which were defined as the sum of each individual's marker effects. RR assumes that all marker effects are sampled from the same normal distribution with zero mean and variance that is estimated by maximum likelihood. With BL, the variance of the marker effect sampling distribution is unique for each marker. This leads to more and less shrinkage on small- and large-effect markers, respectively. We implemented RR in R (R Development Core Team, 2011). The package 'emma' (Kang et al., 2008) was used to estimate the variance components by maximum likelihood. BL was implemented in the R package BLR (de los Campos and Perez Rodriguez, 2010). The parameter values were set to those suggested by Perez et al. 2010 (Pérez et al., 2010). Marker effect estimations were based on 40,000 iterations of sampling after a burn in period of 20,000 iterations. Trace plots of the variance parameters were inspected to check for convergence.

Effect of excluding sparse marker data on the genomic selection accuracy

In order to determine if markers with a large proportion of missing data should be included rather than filtered from the dataset, we assessed the effect of excluding sparse markers, those with a large proportion of missing data points, on the GS

accuracy. Subsets of the NA70 versions of each dataset were used to calculate GS accuracies after imputation with each method. One of the two subsets contained markers that had up to 20% missing data per-marker before imputation, referred to as NA70-sub20. The second subset contained markers that had up to 50% missing data before imputation, referred to as NA70-sub50. The marker set containing markers with up to 70% missing data (which includes all the markers) is referred to as NA70-sub70. For comparison, the original datasets with no simulated missing data were also subsetted so they would contain the same markers as the NA70-sub20 and NA70-sub50, and NA70-sub70 datasets, these marker sets are referred to as NA0-sub20 and NA0-sub50, NA0-sub70. The numbers of markers in each of the marker sets are listed in Table S1, and an example of the marker sets is illustrated in Figure S2. GS accuracy for each marker set-imputation method combination was calculated using RR. For each imputation method, the differences in GS accuracy between versions NA70-sub20, NA70-sub50, and NA70-sub70 were examined to determine how the GS accuracy is affected by including markers with over 20% missing values and with over 50% missing values after applying each imputation method. GS accuracy was also obtained using versions NA0-sub20, NA0-sub50, and NA0-sub70 to determine how the marker subsets affect the GS accuracy when the true genotypic data is known.

Fst calculation

Using each original dataset, individuals were classified into clusters using model based hierarchical agglomerative clustering described by Fraely and Raftery (2002) implemented using the R package 'mclust' (Fraley et al. 2012). The multivariate normal mixture models evaluated were spherical equal volume (EII), spherical unequal volume (VII), diagonal equal volume and shape (EEI), diagonal equal volume, varying shape (EVI), diagonal varying volume, equal shape (VEI), and diagonal varying volume and shape (VVI). The number of clusters evaluated for each model was 1-15. For each dataset the optimal model and number of clusters was chosen according to the Bayesian information criterion (BIC). For all datasets the optimal model was VEI and the optimal number of clusters was 5, 3, 4, and 5 for the WW, SW, DTM, and NAB datasets respectively.

After individuals were classified into clusters, an Fst value for each marker was calculated to determine its level of differentiation due to genetic structure, or in other words, the amount its variance explained by population structure. Fst was calculated as:

$$\mathrm{Fst} = \frac{\overline{p^2} - \bar{P}^2}{\bar{P}(1 - \bar{P})}$$

where $\overline{p^2}$ is the weighted average of the squared allele frequency across subpopulations for one (arbitrary) allele, and $\bar{P}$ is the weighted average allele frequency across the subpopulations for that same allele (Weir and Cockerham, 1984).

RESULTS

Optimal k values for k nearest neighbors and singular value decomposition imputation

The optimal k values for KNNI and SVDI for each dataset and dataset version are listed in Table S1. The optimal k values for KNNI were low, usually between 2 and 4 for most datasets and dataset versions. Compared to the other datasets, optimal k values for the DTM dataset were more than 10 times larger. The optimal KNNI k value for the SRRW dataset version NA70 was also disproportionally higher than the other dataset and dataset versions. The optimal k vales for SVDI varied depending on the dataset and always decreased as the level of missing data increased. It appeared that higher levels of LD between marker pairs and greater numbers of markers favored larger optimal k-values for SVDI.

Effect of excluding sparse marker data on the genomic selection accuracy

For all imputation methods, there was generally a sharp increase in GS accuracy across the NA70-sub20, and NA70-sub50 versions, and a slight increase across the NA70-sub50 and NA70-sub70 versions (Figure S3, panels B-F) indicating that excluding sparse marker data almost always lead to decreased accuracy especially when a more stringent percent missing threshold was used to filter markers. We found that filtering out the same marker sets when the true data was known had an even larger effect on the GS accuracy (Figure S3 panel A), indicating that marker density was a factor limiting the GS accuracy in these populations. Had marker density not been limiting, including sparse markers may not have lead to increased accuracy.

REFERENCES

de los Campos, G., H. Naya, D. Gianola, J. Crossa, A. Legarra *et al.*, 2009 Predicting quantitative traits with regression models for dense molecular markers and pedigree. Genetics 182: 375-85.

de los Campos, G., and P. Perez Rodriguez, 2010 BLR: Bayesian linear regression. R package versión 1.2. Avaliable at: http://CRAN.R-project.org/package=BLR/ (verified 30 July 2012).

Crossa, J., G. de los Campos, P. Pérez, D. Gianola, J. Burgueño *et al.*, 2010 Prediction of genetic values of quantitative traits in plant breeding using pedigree and molecular markers. Genetics 186: 713-724.

Fraley, C., and A.E. Raftery, 2002 Model-based clustering, discriminant analysis, and density estimation. J. Amer. Statist. Assoc. 97: 611-631.

Fraley, C., A.E. Raftery, T.B. Murphey, and L. Scrucca, 2012 mclust Version 4 for R: normal mixture modeling for model-based clustering, classification, and density estimation technical report No. 597, Department of Statistics, University of Washington

Heffner, E. L., J.-L. Jannink, and M. E. Sorrells, 2011 Genomic selection accuracy using multifamily prediction models in a wheat breeding program. Plant Gen. 4: 65-75.

Kang, H. M., N. A. Zaitlen, C. M. Wade, A. Kirby, D. Heckerman *et al.*, 2008 Efficient control of population structure in model organism association mapping. Genetics 178: 1709-1723.

Pérez, P., G. D. L. Campos, J. Crossa, D. Gianola, and G. de los Campos, 2010 Genomic-enabled prediction based on molecular markers and pedigree using the Bayesian linear regression package in R. Plant Genome 3: 106-116.

R Development Core Team, 2011 R: A Language and Environment for Statistical Computing, Vienna. Available at: http://www.r-project.org/ (verified 30 July 2012).

Weir, B. S., and C. C. Cockerham, 1984 Estimating F-statistics for the analysis of population structure. Evolution 6: 1358-1370.

Whittaker, J. C., R. Thompson, and M. C. Denham, 2000 Marker-assisted selection using ridge regression. Genet. Res. 75: 249-252.
